# *NANOGP8*: Evolution of a Human-Specific Retro-Oncogene

**DOI:** 10.1534/g3.112.004366

**Published:** 2012-11-01

**Authors:** Daniel J. Fairbanks, Aaron D. Fairbanks, T. Heath Ogden, Glendon J. Parker, Peter J. Maughan

**Affiliations:** * Department of Biology, Utah Valley University, Orem, Utah 84058; † Department of Plant and Wildlife Sciences, Brigham Young University, Provo, Utah 84602

**Keywords:** pseudogene evolution, retroelement, SVA element, cancer, human diversity

## Abstract

*NANOGP8* is a human (*Homo sapiens*) retrogene, expressed predominantly in cancer cells where its protein product is tumorigenic. It arose through retrotransposition from its parent gene, *NANOG*, which is expressed predominantly in embryonic stem cells. Based on identification of fixed and polymorphic variants in a genetically diverse set of human *NANOG* and *NANOGP8* sequences, we estimated the evolutionary origin of *NANOGP8* at approximately 0.9 to 2.5 million years ago, more recent than previously estimated. We also discovered that *NANOGP8* arose from a derived variant allele of *NANOG* containing a 22-nucleotide pair deletion in the 3′ UTR, which has remained polymorphic in modern humans. Evidence from our experiments indicates that *NANOGP8* is fixed in modern humans even though its parent allele is polymorphic. The presence of *NANOGP8*-specific sequences in Neanderthal reads provided definitive evidence that *NANOGP8* is also present in the Neanderthal genome. Some variants between the reference sequences of *NANOG* and *NANOGP8* utilized in cancer research to distinguish RT-PCR products are polymorphic within *NANOG* or *NANOGP8* and thus are not universally reliable as distinguishing features. *NANOGP8* was inserted in reverse orientation into the LTR region of an SVA retroelement that arose in a human-chimpanzee-gorilla common ancestor after divergence of the orangutan ancestral lineage. Transcription factor binding sites within and beyond this LTR may promote expression of *NANOGP8* in cancer cells, although current evidence is inferential. The fact that *NANOGP8* is a human-specific retro-oncogene may partially explain the higher genetic predisposition for cancer in humans compared with other primates.

The human (*Homo sapiens*) genome contains nearly three million copies of retroelements—segments of DNA derived from reverse transcription of RNA—constituting more than 40% of the genome (International Human Genome Sequencing Consortium 2001). These retroelements are classified as long interspersed nuclear elements (LINEs), short interspersed nuclear elements (SINEs), LTR elements, and retropseudogenes (also known as processed pseudogenes). The number of genomic retroelements has been increasing over evolutionary time through retrotransposition, by which an existing segment of DNA is transcribed in cells of the germline, the resulting RNA is reverse transcribed, and the derived DNA is inserted into the genome. From that point forward, the retrotransposed element at its new location in the DNA is heritable and may eventually become fixed in the genome of a species, while its parent sequence remains at its original site in the genome.

Retroelements are especially abundant in human and great ape genomes, due in part to retrotranspositional bursts that predate the evolutionary divergence of their ancestral lineages. As a consequence, more than 99% of retroelements in the human and chimpanzee (*Pan troglodytes*) genomes are orthologous, although recent retrotranspositional activity has generated thousands of lineage-specific retroelements in these two species (Chimpanzee Sequencing and Analysis Consortium 2005).

Some full-length LINEs are autonomous retroelements, containing functional genes that encode all of the products necessary for their own retrotransposition. These products do not act solely on LINEs but may retrotranspose RNA molecules transcribed in the germline from various segments of DNA—including LINEs, SINEs, LTR elements, and genes—progressively increasing the number of retroelements in the genome. Over evolutionary time, as retroelements become fixed in the genome of a species, those located in or near a gene may exert a functional role in expression of that gene (Wang *et al.* 2005).

More than 8000 retropseudogenes in the human genome are derived from protein-encoding parent genes (Zhang *et al.* 2003) and can be readily identified by their similarity to the mRNAs encoded by those genes—they lack the gene’s introns and often have a remnant of a 3′ poly(A) tail—and by short target-site duplications (TSDs) at their borders. A significant number of genes have generated retropseudogene families consisting of multiple copies derived from the same parent gene. For example, 22.8% of all retropseudogenes in the human genome are members of ribosomal protein pseudogene families, each with numerous members. Protein-encoding cellular housekeeping genes, such as *CYCS* and *GADPH*, have likewise generated large pseudogene families (Zhang *et al.* 2003; Liu *et al.* 2009).

Most retropseudogenes have undergone extensive mutational decay over evolutionary time, acquiring mutations that render them non-functional, such as large deletions and insertions (including retroelement insertions), frameshifts, and premature termination codons. Such mutations accumulate because retropseudogenes typically fail to function as genes from the time of their origin and are thus free from constraints of purifying selection. Some evolutionarily recent retropseudogenes lack any disabling mutations but are nonetheless transcriptionally quiescent because they are removed from the promoter and transcription-enhancing sequences of their parent gene. A relatively small number of retropseudogenes can be classified as retrogenes because they are transcribed and translated into functional proteins, often in different cell types and under different transcriptional controls than their parent gene (Rohozinski *et al.* 2012; Ishiguro *et al.* 2012; Ma *et al.* 2011; Snider *et al.* 2010).

The *NANOG* gene encodes a homeobox transcription factor that plays an essential role in maintaining pluripotency in vertebrate embryonic stem cells (Mitsui *et al.* 2003; Chambers *et al.* 2003; Chambers *et al.* 2007; Wang *et al.* 2006; Tay *et al.* 2008). Its pseudogene family in the human genome consists of 11 members, named *NANOGP1* through *NANOGP11*, *NANOGP1* being a single duplication pseudogene, and *NANOGP2* through *NANOGP11*, 10 retropseudogenes (Booth and Holland 2004). Booth and Holland (2004) determined the relative evolutionary ages of the *NANOG* pseudogenes based on substitutional divergence from the *NANOG* parent gene and concluded that *NANOGP8* is the most recent. Fairbanks and Maughan (2006) reported that the human and chimpanzee genomes share all *NANOG* pseudogenes at orthologous chromosomal positions except for *NANOGP8*, which is absent from the chimpanzee genome. *NANOGP8* therefore arose in the hominin ancestral lineage after the hominin-panin (human-chimpanzee) ancestral divergence and is a lineage-specific retropseudogene unique to humans among extant species.

In addition to its presence in embryonic stem cells, the NANOG protein is present in cancer stem cells (CSCs) in a variety of human cancers. Two distinct but related NANOG proteins may be active in CSCs, one encoded by *NANOG* and the other by *NANOGP8*, which is transcriptionally active as a retrogene in several types of cancer cells (Zhang *et al.* 2006; Jeter *et al.* 2009, 2011; Zbinden *et al.* 2010; Ambady *et al.* 2010; Eberle *et al.*, 2010; Zhang *et al.* 2010, Ma *et al.* 2011, 2012; Ishiguro *et al.* 2012; Uchino *et al.* 2012; Ibrahim *et al.* 2012). Although both the *NANOG* gene and the *NANOGP8* retrogene may be expressed in CSCs, experimental evidence suggests that CSCs preferentially express *NANOGP8* and that the NANOGP8 protein promotes tumorigenesis more readily than NANOG (Jeter *et al.* 2009; Zbinden *et al.* 2010; Jeter *et al.* 2011; Uchino *et al.* 2012). Importantly, knockdown of *NANOG*/*NANOGP8* mRNA in cancer cells inhibits tumorigenesis and clonogenic growth of breast, colon, prostate, and gastrointestinal cancer cell lines (Jeter *et al.* 2009; Jeter *et al.* 2011; Uchino *et al.* 2012). These discoveries confirm a key role for *NANOGP8* in tumorigenesis and suggest that suppression of *NANOGP8* gene expression or protein activity may potentially be developed as a treatment for cancer.

The cancer rate in humans exceeds that of wild and captive great apes, and this difference is probably due in part to genetic differences (McClure 1973; Beniashvili 1989; Waters *et al.* 1998; Puente *et al.* 2006). In an attempt to determine the genetic basis of this discrepancy, Puente *et al.* (2006) examined 333 genes known to influence the incidence and progression of cancer, comparing them in the human and chimpanzee genomes. They determined that all of these genes are present at orthologous chromosomal positions, contain open reading frames, and are highly conserved in both species. *NANOGP8*, however, had not been identified as a cancer-promoting retrogene at the time of their study and was not included in it. Several retrogenes are known to influence the incidence and progression of cancer (Rohozinski *et al.* 2012; Young *et al.* 2010; Liu *et al.* 2009). However, to date, *NANOGP8* is the only known cancer-promoting retrogene that is exclusive to humans and thus may be partially responsible for the higher predisposition for cancer in humans compared with other primates.

In the research reported here, we document the evolutionary history of *NANOGP8*, distinguish polymorphic from fixed variants between *NANOGP8* and *NANOG*, and use a comparative genomic approach to identify a series of retrotranspositional and mutational events that allowed *NANOGP8* to evolve as a human-specific retro-oncogene.

## Materials and Methods

### DNA samples

Human DNA samples were obtained from the Coriell Institute for Medical Research (Camden, NJ). Samples were from the Human Variation Panel, including 110 individuals in the SNP500Cancer panel (Packer *et al.* 2004) and 9 individuals in the Africans South of the Sahara panel.

### PCR amplification and identification of amplified fragments

PCR amplification of DNA fragments specific to *NANOG* or *NANOGP8* from genomic DNA is complicated by the exceptionally high similarity between *NANOG* and *NANOGP8*, and their similarity to other *NANOG* pseudogenes in the human genome. These sequence similarities substantially limit identification of appropriate primer-binding sites because all but a few sites result in co-amplification of unintended non-target fragments from one or more paralogous sequences, often the same size as the target fragment. To preclude unintended amplification, we selected primer pairs that target sites unique to *NANOG* and/or *NANOGP8*, excluding all other *NANOG* pseudogenes. In some cases, primers pairs co-amplified fragments from both *NANOG* and *NANOGP8*, but fragments were differentiated by size due to the presence of introns in fragments amplified from *NANOG* and their absence in those amplified from *NANOGP8*. Predicted fragment sizes, primer targeting, and preclusion of unintended amplification were verified by Primer-BLAST (http://www.ncbi.nlm.nih.gov/tools/primer-blast).

Table 1 lists the primers used for PCR amplifications from genomic DNA, their target sites, primer pairings, and the sources and sizes of amplified fragments for these pairings. All primers were manufactured by Integrated DNA Technologies (Coralville, IA). PCR amplification was accomplished using Qiagen HotStarTaq Plus Master Mix (Valencia, CA) according to the manufacturer’s recommendations. The amplification protocol consisted of an initial denaturation step of 5 min at 95°, followed by 35 cycles of amplification consisting of 30 s denaturation at 94°, 30 s for primer annealing at 62°, and from 0.5 to 2 min of extension at 72°, depending on the expected amplification product size (1 min/1 kb). PCR products were separated on 1% agarose gels run in 0.5X TBE and visualized by ethidium bromide staining and UV transillumination. Special care was taken during PCR setup to avoid extraneous human DNA contamination, including the use of clean rooms and DNA-free reagents and tubes. Controls lacking template DNA were included with each experiment.

**Table 1:** Primers for PCR amplification and sequencing, primer binding sites, primer pairings, and amplified fragment sizes and sites

Primer		Binding Site*^a^*
F1: 5′CAAAGCACATCTTGCCAGGA3′		c.–212 to c.–193 (5′ insertion site of *NANOGP8*)
F2: 5′GGCCGAAGAATAGCAATGGTGTGACG3′		c.473 to c.498 (exon 3 in *NANOG* and *NANOGP8*)
F3: 5′CTCCAGTCACAGACAGTTCTGGTTGTCC3′		c.502–64C to c.502–37C (intron 3 in *NANOG*)
F4: 5′GAATAGCAATGGTGTGACGCAGAAGG3′		c.477 to c.496 (splice site for exons 3 and 4 in *NANOGP8*)
F5: 5′GGACAGCCCTGATTCTTCCACCAG3′		c.189 to c.212 (second exon in *NANOGP8*, internal primer for sequencing cloned DNA)
R1: 5′GGTTATTAAAATGTCTTTTCTAGGCAGGGCGC3′		c.*512 to c.*543 (3′ boundary of *Alu* element in 3′ UTR of *NANOG* and *NANOGP8*)
R2: 5′CTTATCTATAGCCAGAGACGGCAGCC3′		c.*546 to c.*551 and c.*574 to c.*593 (22 nucleotide-pair deletion in 3′ UTR of *NANOG* and *NANOGP8*)
R3: 5′GCTTCTATCAATGTTGTCCTTAGC3′		c.*550 to c.*573 (ancestral, non-deletion site in *NANOG* 3′ UTR)
R4: 5′CCATACTCCACCCTCCATGAG3′		c.*140 to c.*160 (3′ UTR in *NANOGP8*, internal primer for sequencing cloned DNA)
		
**Primer Pair**	**Fragment Size**	**Fragment Site*****a***
F1/R1	1681	c.–212 to c.*543 from *NANOGP8*
F2/R1	1132	c.473 to c.*543 from *NANOG*
	997	c.473 to c.*543 from *NANOGP8*
F2/R2	1157	c.473 to c.*593 from *NANOG* deletion allele
	1025	c.473 to c.*593 from *NANOGP8*
F2/R3	1154	c.473 to c.*573 from NANOG allele without deletion
F3/R1	1029	c.502–64C to c.*543 from *NANOG*
F4/R1	990	c.477 to c.*543 from *NANOGP8*

a Nucleotides are numbered in accordance with Nomenclature for the Description of Sequence Variants of the Human Genome Variation Society (http://www.hgvs.org/mutnomen), as follows: The symbol “c.” refers to coding sequence. Numbering in the reading frame begins at the first nucleotide of the initiation codon and ends with the final nucleotide of the termination codon. Nucleotides in the 5′ UTR are numbered in reverse, denoted with a negative sign (–), with the nucleotide preceding the first nucleotide of the reading frame designated as –1. Nucleotides in the 3′ UTR are numbered consecutively, denoted with an asterisk (^*^), with the first nucleotide beyond the final nucleotide of the reading frame designated as ^*^1.

### DNA sequencing

A 1681 bp PCR fragment consisting of 78% of the complete sequence of *NANOGP8*, including the entire 5′ UTR and reading frame and the 3′ UTR between the termination codon and an *Alu* element insertion, was amplified with primer pair F1/R1 (Table 1) from 10 geographically diverse individuals selected from the Coriell SNP500Cancer and Africans South of the Sahara panels (supporting information, File S1). PCR fragments of the target size amplified from these 10 individuals were cloned using the pGEM-T Easy Vector System II (Promega, Madison, WI). Recombinant clones were identified by standard blue/white screening methods with IPTG and X-Gal. Plasmid DNA from each selected recombinant clone was purified using GenElute plasmid miniprep Kit (Sigma, St. Louis, MO). Isolated plasmid DNA was sequenced bidirectionally using standard M13 (F/R) primers and internal primers F4 and R5 (Table 1).

A 1029 bp PCR product specific to *NANOG*, amplified with primer pair F3/R1 (Table 1), and a 990 bp PCR product specific to *NANOGP8*, amplified with primer pair F4/R1, were purified using a standard ExoSAP (exonuclease I/shrimp alkaline phosphatase) protocol and single-pass sequenced directly from PCR products using the F3 primer for *NANOG* and the F4 primer for *NANOGP8*.

### DNA sequencing, assembly, and alignment

DNA sequencing was performed at the Brigham Young University DNA Sequencing Center (Provo, UT) using standard ABI Prism Taq dye-terminator cycle-sequencing methodology. DNA sequences were analyzed and assembled using Geneious software (Biomatters, Auckland, New Zealand), and aligned with either megablast (http://www.ncbi.nlm.nih.gov) or MEGA 5.05 for Mac OS X (http://www.megasoftware.net/megamac.php) (Tamura *et al.* 2011).

### Nucleotide numbering and DNA variant nomenclature

Nucleotide numbering and DNA-variant designations were assigned in accordance with Nomenclature for the Description of Sequence Variants of the Human Genome Variation Society (http://www.hgvs.org/mutnomen), with the following two clarifications: (1) Guidelines specify that the largest transcript of a gene should be used. Accordingly, we used the coding sequence of the alternate reference assembly of *NANOG* with introns removed (AC_000144.1 [gi 157704453]: 7755619.0.7762294). To maintain consistent numbering in *NANOG* and *NANOGP8*, all nucleotides in *NANOGP8* were numbered according to their alignment with the inferred mRNA sequence in the alternate reference assembly for *NANOG*. Variations in numbering are relevant only to the 3′ UTR where deletions and insertions are present in some sequences. (2) To accurately reflect mutational evolution, all substitution variants within *NANOG* or *NANOGP8*, or between the two, are designated with the ancestral nucleotide first, followed by the derived nucleotide (*e.g. G > A*), regardless of which nucleotide is present in any particular reference sequence.

## Results and Discussion

### The origin of *NANOGP8* is evolutionarily more recent than previously estimated

**Figure 1:**
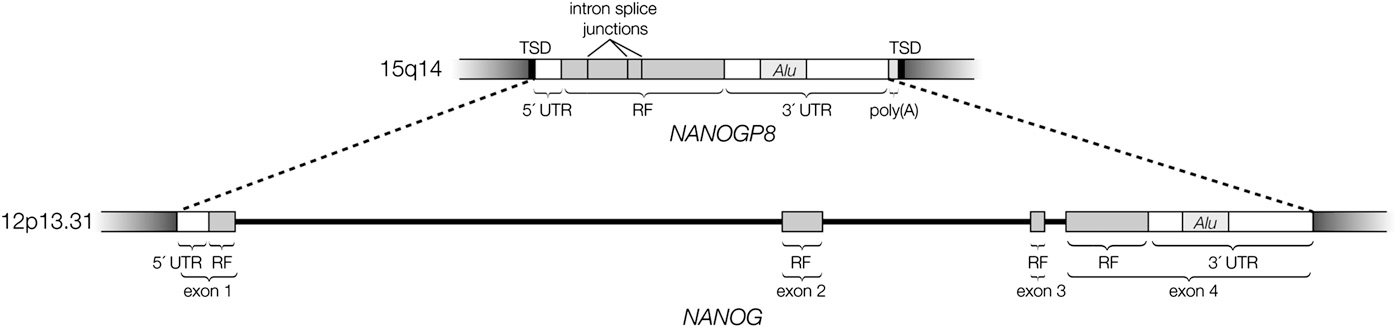
Comparison of *NANOGP8* with its parent gene *NANOG*. 3′ UTR, 3′ untranslated region; 5′ UTR, 5′ untranslated region; *Alu*, *Alu* element in 3′ UTR; RF, reading frame; TSD, target site duplication.

*NANOGP8* resides on the long arm of human chromosome 15 in a relatively gene-free region (15q14). As depicted in Figure 1, *NANOGP8* is bordered on both ends by a target-site duplication of 15 nucleotide pairs, and it lacks all three introns that are present in *NANOG*. It has a 5′ UTR of 197 nucleotide pairs, an intact reading frame of 918 nucleotide pairs, a 3′ UTR of 967 nucleotide pairs, including an *Alu* element, and a poly(A) tail in the current human primary and alternate reference assemblies (NC_000015.9 [gi 224589806]: c35375427 . . 35377509, AC_000147.1 [gi 157709134]: c12222739 . . 12220659).

As Booth and Holland (2004) pointed out, the *Alu* element in the 3′ UTR of *NANOGP8* is also present in the *NANOG* parent gene but is absent from all other human *NANOG* pseudogenes, evidence of a relatively recent evolutionary origin for this *Alu* element and a more recent origin for *NANOGP8*. Fairbanks and Maughan (2006) found this *Alu* element in the chimpanzee *NANOG* gene and also found it to be absent in all chimpanzee *NANOG* pseudogenes. Using the current human *NANOG* mRNA reference sequence as a query (NM_024865.2 [gi: 153945815]), we searched the Sumatran orangutan (*Pongo abelii*) and rhesus macaque (*Macaca mulatta*) assembled genomes and the gorilla (*Gorilla gorilla*) whole-genome shotgun sequences for this *Alu* element, and we found it to be present in the gorilla *NANOG* gene but absent from the orthologous site in the orangutan and rhesus macaque *NANOG* genes. It therefore was inserted into *NANOG* in the human-chimpanzee-gorilla common ancestral lineage between 8 and 16 million years ago, after the divergence of the orangutan ancestral lineage (Locke *et al.* 2011). Because this *Alu* element is present exclusively in *NANOG* and *NANOGP8* and absent in all other *NANOG* pseudogenes, we used its 3′-insertion site as a primer-binding site (primer R1, Table 1) in several of our PCR experiments to exclude unintended amplification from other *NANOG* pseudogenes.

*NANOGP8* is highly similar to its parent *NANOG* gene’s mRNA. Booth and Holland (2004), Jeter *et al.* (2009), and Uchino *et al.* (2012) aligned *NANOG* and *NANOGP8* reference sequences within the reading frame and identified variants between them to estimate the evolutionary age of *NANOGP8* or to distinguish *NANOG* and *NANOGP8* RT-PCR products in cancer cells. Of the eight reading-frame variants between *NANOG* and *NANOGP8* identified in these studies, only two (*c.144G > A* and *c.759G > C*) were common to all three studies, indicative of modern polymorphisms in *NANOG* or *NANOGP8*. Identification of modern polymorphisms is important for molecular-clock analysis because their inclusion as presumably fixed substitutions could overestimate the age of *NANOGP8*. Moreover, reliance on variants between reference sequences to distinguish *NANOG* and *NANOGP8* RT-PCR products in cancer research may result in misidentification of these products if a variant is a modern polymorphism within either *NANOG* or *NANOGP8*.

To initially identify modern polymorphisms within *NANOG*, we aligned the primary and alternate reference assemblies for this gene throughout the full length of the transcribed region, excluding introns (primary reference assembly NC_000012.11 [gi 22458903]: 7941995 . . 7948655, alternate reference assembly AC_000144.1 [gi 157704453]: 7755619 . . 7762294). We found a surprisingly high number of variants between these two reference sequences, suggesting a high degree of modern polymorphism within *NANOG*: 16 substituted and 27 deleted nucleotides within the 2115 nucleotides of the mRNA. The degree of variation between these two *NANOG* reference sequences within the reading frame is equal to the presumably fixed variation previously reported between *NANOG* and *NANOGP8* in the same region (Booth and Holland 2004; Jeter *et al.* 2009), calling into question the accuracy of the previous age estimate of 5.2 million years ago for the origin of *NANOGP8* (Booth and Holland 2004).

Six of the substitutions we identified between the *NANOG* reference sequences are in the reading frame: *c.165C > T*, *c.246G > T*, *c.276G > A*, *c.363C > T*, *c.531C > T*, and *c.798C > T* (Table 2). All six have been identified as modern polymorphisms in the current NCBI dbSNP Report for *NANOG* (http://www.ncbi.nlm.nih.gov/SNP/snp_ref.cgi?locusId=79923), with relatively high minor allele frequencies (MAF) ranging from 0.1836 to 0.3863 (rs4294629, rs2889551, rs4354764, rs4438116, rs4012939, and rs4012937). All six are in the third nucleotide of their respective codons, and only one is non-synonymous (*c.246T > G p.Asn82Lys*, rs2889551). Booth and Holland (2004) experimentally confirmed this latter substitution variant as a modern polymorphism in *NANOG* through direct sequencing. The remaining 10 substitutions are in the 3′ UTR, as are all 27 deletions. Of those deletions, 22 are contiguous as a single deleted segment downstream of the 3′ border of the *Alu* element (c.*552_*573del), and 4 are variations within poly-T mononucleotide repeats [c.*184T(12–15) and c.*223T(18–19)]. The remaining deletion is a single nucleotide within the 3′ UTR *Alu* element (c.*496del).

**Table 2:** Variants between *NANOG* and *NANOGP8* sequences detected by comparison of current primary and alternate reference assemblies and sequences we obtained experimentally

Variant in Coding DNA*^a^*	Variant in Protein*^a^*	*NANOG*	*NANOGP8*
*–135T > C*	—	T	T/C*^b^*
*47C > A*	*Ala16Glu*	C	C/A
*126T > C*	=	T	T/C
***144G > A***	**=**	**G**	**A**
*165T > C*	=	T/C	T
*190G > T*	*Asp64Tyr*	G	G/T*^b^*
*246T > G*	*Asn82Lys*	T/G	T
*276G > A*	=	G/A	G
*363C > T*	=	C/T	C
*531C > T*	=	C/T	C
*552A > T*	=	A	A/T*^b^*
*629C > T*	*Thr210Ile*	C	C/T*^b^*
*754A > C*	*Met252Leu*	A	A/C*^b^*
***759G > C***	***Gln253His***	**G**	**C**
*798C > T*	=	C/T	C
*916–917del*	*Ter309fs*	TG	TG/del*^b^*
*7G > A	—	G	G/A*^b^*
*44G > A	—	G	G/A*^b^*
*184T(12–17)	—	poly(T)	poly(T)
*223T(17–20)	—	poly(T)	poly(T)
*243G > A	—	G/A	G
*310T > C	—	T/C	T
*313C > G	—	C	C/G
*315C > T	—	C	C/T
*413G > A	—	G/A	G
*467G > A	—	G	G/A
*496del	—	T/del	T
*512G > A	—	G	G/A
*552–*573del	—	=/del	del
*606T > G	—	**T**	**G**
*663A > G	—	A/G	A
*802C > T	—	C/T	C
*843C > T	—	C/T	C
*875G > A	—	G/A	A
*910C > A	—	C/A	C
*956A > G	—	A/G	A

Polymorphic variants are indicated with a forward slash separating the ancestral and derived variants, with the ancestral variant indicated first. All variants are polymorphic in either *NANOG* or *NANOGP8* except for three, *144G > A*, *759G > C*, and ^*^606T > G, indicated in boldface.

a In accordance with human genetic nomenclature guidelines for designating DNA variants in genes (http://www.hgvs.org/mutnomen), nucleotides in the reading frame are numbered relative to the first nucleotide in the ATG initiation codon; positions preceded by a negative (–) sign are in the 5′ UTR and are numbered in reverse relative to the first nucleotide in the ATG initiation codon; and positions indicated with an asterisk (^*^) are in the 3′ UTR relative to the first nucleotide beyond the termination codon. The symbol “=” denotes that a nucleotide substitution has no effect on the protein (*i.e.* a synonymous variant in the reading frame).

b Variants at these sites were not present in the comparison of primary and alternate reference assemblies of *NANOGP8* or in other publications. However, we observed polymorphism in at least two individuals for each of these sites in the sequences we obtained.

We also aligned the sequences for *NANOGP8* from the current primary and alternate reference assemblies (primary reference assembly NC_000015.9 [gi 224589806]: c35375427 . . 35377509, alternate reference assembly AC_000147.1 [gi 157709134]: c12222739 . . 12220659). The number of variants between these *NANOGP8* sequences was considerably fewer than in *NANOG*: four substitution variants, all in the 3′ UTR (c.*75C > T, c.*313C > G, c.*315C > T, and c.*512G > A), and variation in one of the two poly(T) mononucleotide repeats of the 3′ UTR [c.*223T(18–20)].

Alignment of the human *NANOG* and *NANOGP8* reference sequences with other *NANOG* pseudogenes, with the *NANOG* gene in the assembled chimpanzee, orangutan, and rhesus macaque genomes, and with the gorilla *NANOG* sequence from a WGS contig (gi 269709276) allowed us to classify all substitution variants within and between *NANOG* and *NANOGP8* as ancestral or derived. We were unable to determine which mononucleotide repeat polymorphisms are ancestral in the two poly(T) segments in the 3′ UTR because they vary in repeat number within and among species, as expected for mononucleotide repeats. A 22-nucleotide pair deletion in the 3′ UTR (c.*552_*573del) is present only in *NANOG* and *NANOGP8* and is exclusive to humans; it thus is derived. With the exception of this deletion and the poly(T) mononucleotide repeats in the 3′ UTR, *NANOGP8* carries the ancestral nucleotides for all variants between the two reference sequences of *NANOG*, and likewise, *NANOG* carries the ancestral sequence for all variants between the two reference sequences of *NANOGP8*. From these observations, we concluded that the ancestral nucleotides for all substitution variants between the two reference sequences of *NANOG* and between the two reference sequences of *NANOGP8* were present in the parent allele of *NANOG* at the time of *NANOGP8*’s origin.

On the basis of this conclusion, we merged the primary and alternate reference sequences for *NANOG* by converting all substitution variants between the two to the ancestral form and did the same for *NANOGP8*, and then we aligned these converted *NANOG* and *NANOGP8* sequences. This comparison reduced the number of potentially fixed substitution variants between *NANOG* and *NANOGP8* to four: three within the reading frame (*c.47C > A*, *c.144G > A*, and *c.759G > C*) and one in the 3′ UTR (*606T > G). In all four cases, *NANOG* carries the ancestral nucleotide, and the derived variant is in *NANOGP8* (Table 2). This analysis, therefore, allowed us to reconstruct the sequence of the *NANOG* parent allele at the time of *NANOGP8*’s origin, permitting a more accurate dating of this origin.

To identify additional modern polymorphisms in *NANOGP8* and to obtain further evidence of potentially fixed variants, we used primer pair F1/R1 to amplify 78% of the complete sequence of *NANOGP8*, including the entire 5′ UTR and reading frame and the first 511 nucleotides of the 3′ UTR, from 10 geographically diverse individuals. Then we cloned these PCR fragments and obtained high-quality sequences of the complete clones (GenBank accession nos. JX104830–JX104848, supporting information, File S1). To more extensively examine a particular region in the fourth exon of *NANOG* and *NANOGP8*, which contains an important variant (*c.759G > C p.Gln253His*, described in detail later), we amplified a 1029 nucleotide-pair fragment from exon 4 of *NANOG* with primer pair F3/R1 (Table 1) and a 990 nucleotide-pair fragment from the same region of *NANOGP8* with primer pair F4/R1 (Table 1) from 94 geographically diverse individuals. We then conducted single-pass sequencing of these PCR products, obtaining approximately 590 nucleotides of reliable sequence from the 5′ end of exon 4 into the first poly(T) segment of the 3′ UTR (GenBank accession nos. JX104849–JX104942 for *NANOGP8*, and JX104943–JX105036 for *NANOG*, File S2 and File S3).

We found that the *c.47C > A* variant is polymorphic and apparently rare in *NANOGP8* (see File S1), as suggested by Jeter *et al.* (2009), reducing the number of evidently fixed variants between *NANOG* and *NANOGP8* to three: *c.144G > A*, *c.759G > C*, and c.*606T > G. The latter of these variants lies near the end of the 3′ UTR, outside of the region we sequenced, and thus, we could not confirm whether it is fixed or polymorphic. All evidence of its fixation is derived from four *NANOGP8* sequences that include the entire 3′ UTR in the NCBI nucleotide database; no other *NANOGP8* sequences in the NCBI nucleotide database contain this region. We therefore have focused our analysis on the two evidently fixed variants within the reading frame: *c.144G > A* and *c.759G > C*.

Table 2 summarizes all variants we found between *NANOG* and *NANOGP8* by aligning different reference sequences and in the sequences we obtained experimentally, identifying those variants confirmed to be modern polymorphisms in either *NANOG* or *NANOGP8*. All variants between the *NANOG* and *NANOGP8* reading frames identified in previous publications (Booth and Holland 2004; Zhang *et al.* 2006; Jeter *et al.* 2009; Uchino *et al.* 2012) were evident in the sequences we obtained, all but two of them modern polymorphisms.

Of the two evidently fixed variants between *NANOG* and *NANOGP8* within the reading frame, one is synonymous (*c.144G > A*), and one is non-synonymous (*c.759G > C p.Glu253His*). Sequencing of PCR products from exon 4 of *NANOG* and *NANOGP8* in DNA samples from 94 geographically diverse individuals demonstrated that the ancestral G at c.759 in *NANOG* is homozygous in all individuals tested and that the derived C at this site in *NANOGP8* is homozygous in all individuals tested, strong evidence that this variant is an ancient mutation in *NANOGP8* and a fixed variant that reliably distinguishes *NANOG* and *NANOGP8*. Therefore, the proteins encoded by *NANOG* and *NANOGP8* differ by only a single fixed amino acid substitution, although modern polymorphisms in both *NANOG* and *NANOGP8* alter other amino acids in some individuals.

Presumably we could have utilized SNP databases for *NANOG* to identify additional polymorphisms. However, our analysis of variants between *NANOG* and *NANOGP8* suggests that SNP databases for *NANOG* are prone to error due to misassignment of short-read sequences, a consequence of the high similarity of paralogous sequences in *NANOG* and its pseudogenes. For example, the current NCBI dbSNP report for *NANOG* has incorrectly assigned the two evidently fixed variants in *NANOGP8* to *NANOG* as modern polymorphisms (rs2377097 for *c.144G > A*, and rs4012938 for *c.759G > C*), as well as several other variants that, according to our review of sequences, belong to *NANOGP8* or other *NANOG* pseudogenes.

Two independent lines of evidence from our analysis allowed us to estimate the evolutionary age of *NANOGP8*. First, evidence that *NANOGP8* is present in the Neanderthal genome (which we will discuss momentarily) and evidence published by Fairbanks and Maughan (2006) that it is absent from the chimpanzee genome indicate that it must have originated before the human-Neanderthal divergence and after the hominin-panin divergence. This observation allowed us to establish lower and upper boundaries for its origin, independent of any variants it carries relative to its parent gene. The lower boundary is the human-Neanderthal divergence, which Green *et al.* (2008) estimated as 520,000 to 800,000 years ago, based on mitochondrial genome analysis, whereas Green *et al.* (2010) estimated it as between 270,000 and 440,000 years ago, based on nuclear genome-wide analysis. The upper boundary is the hominin-panin divergence, which was recently estimated as 5.5–7 million years ago, based on a synthesis of genomic and fossil data (Scally *et al.*, 2012).

Second, inferred substitution rates for human retropseudogenes can be used to estimate the origin of *NANOGP8* by identifying fixed variants in *NANOGP8* relative to the sequence of the *NANOG* parent allele at the time of *NANOGP8*’s origin. Such an estimate is based on the assumption that substitutions in *NANOGP8* are selectively neutral and that fixed variants accumulate in *NANOGP8* at the same rate as in other autosomal human retropseudogenes. Although there is strong evidence that *NANOGP8* is a retrogene expressed in cancer cells, there is no evidence in our data or in previous publications to contradict the assumption that variants in *NANOGP8* are selectively neutral. Therefore, in line with Booth and Holland’s (2004) assumption, we have treated *NANOGP8* as a neutrally evolving retropseudogene for estimation of its evolutionary age.

According to a recent review (Keightly 2012), the most rigorous analysis of autosomal substitution rates in human retropseudogenes was that conducted by Nachman and Crowell (2000), which Booth and Holland (2004) combined with results of Martinez-Arias *et al.* (2001) to derive a rate of 1.25 × 10^−9^ substitutions fixed per site per year. Utilizing this rate, Booth and Holland (2004) estimated the origin of *NANOGP8* as 5.2 million years ago (six variants across 915 sites in the reading frame, excluding the termination codon). Our analysis reduced the number of evidently fixed variants to two (*c.144G > A* and *c.759G > C*) and extended the sequenced region to 1622 nucleotides, which, based on this substitution rate, reduces the estimated origin of *NANOGP8* to approximately one million years ago.

Nachman and Crowell (2000) and Keightly (2012) pointed out that inferred substitution rates for human retropseudogenes are dependent on assumptions regarding the ancestral effective population size and the time of the hominin-panin divergence. By varying these parameters, Nachman and Crowell (2000) listed several possible rates, ranging from 0.65 × 10^−9^ to 1.35 × 10^−9^ per year, and Keightly (2012) suggested two rates: 1.1 × 10^−9^ per year and 0.5 × 10^−9^ per year. The former of these is similar to the rate proposed by Booth and Holland (2004), and the latter is consistent with a genomic mutation rate of (0.5–0.6) × 10^−9^ per year for modern humans, as summarized by Scally *et al.* (2012). If we apply the extremes of the rates proposed by Keightly (2012) and Nachman and Crowell (2000) as a range (1.35 × 10^−9^ to 0.5 × 10^−9^ per year), the origin of *NANOGP8* falls between 0.9 and 2.5 million years ago, still considerably less than previously estimated.

### A derived 22 nucleotide-pair deletion is polymorphic in *NANOG* but monomorphic in *NANOGP8*

*NANOGP8* contains a derived 22 nucleotide-pair deletion in the 3′ UTR (c.*552_*573del). Jeter *et al.* (2009, 2011) considered this deletion to be a variant that distinguishes *NANOGP8* from *NANOG* based on the human genome primary reference assembly available at the time, and they relied on it as a primer-binding site to differentially amplify *NANOG* and *NANOGP8* qRT-PCR products from cancer cells, as did Ma *et al.* (2011, 2012) and Ibrahim *et al.* (2012). However, in the current human genome primary reference assembly, both *NANOG* and *NANOGP8* contain this deletion, indicating that it is not a universally reliable distinguishing feature. The *NANOG* sequence in the alternate reference assembly does not carry this deletion, evidence that the deletion is polymorphic in *NANOG* among modern humans, as noted by Zbinden *et al.* (2010).

The presence of this deletion in *NANOGP8* suggests that the deletion arose in *NANOG* prior to the origin of *NANOGP8* and that *NANOGP8* then arose from the *NANOG* allele containing the deletion. Our observation that this deletion is derived and polymorphic in modern humans offers further evidence that *NANOGP8*’s origin must be relatively recent because a more ancient deletion in the *NANOG* parent allele should have become fixed or lost. Initially, this observation that *NANOGP8*’s parent allele is derived and polymorphic suggested to us that the presence/absence of *NANOGP8* might also be polymorphic among modern humans.

To test this possibility, we utilized primer pair F2/R1 to simultaneously amplify different-sized fragments from *NANOG* and *NANOGP8* (Table 1) to screen for the possible presence/absence of *NANOGP8* in 119 geographically diverse individuals from the entire Coriell SNP500Cancer and Africans South of the Sahara panels. This experiment, as well as subsequent experiments with primer pairs F2/R2 and F4/R2 (Table 1), demonstrated that all individuals in both panels uniformly carry *NANOGP8*, evidence that *NANOGP8* originated in Africa and was fixed there prior to the migrations approximately 60,000 years ago that founded the first *Homo sapiens* populations outside of Africa.

We screened all individuals in these two panels for the presence/absence of the 22 nucleotide-pair deletion (c.*552_*573del) in both *NANOG* and *NANOGP8* with primer pairs F2/R2 and F2/R3 (Table 1). The deletion was uniformly present in *NANOGP8* in all individuals, but both the ancestral (=) and deletion (*552–*573del) alleles in *NANOG* were highly polymorphic and distributed in populations throughout the world, with no evident geographic pattern for their distribution (Table 3).

**Table 3:** Distribution of the ancestral (=) and deletion (*552–*573del) alleles in the 3′ UTR of *NANOG* among 119 geographically diverse individuals

Population	Number of Individuals Tested	Homozygous for Ancestral (=) Allele*^a^*	Heterozygous	Homozygous for Deletion (*552–*573del) Allele*^b^*
Africans South of the Sahara	9	1	6	2
Biaka Pygmy	4	1	2	1
Mbuti Pygmy	5	2	1	2
African-American	14	2	8	4
Druze	5	5	0	0
Indo-Pakistani	3	3	0	0
Russian Krasnodar	3	2	1	0
Ami	5	0	2	3
Chinese	5	1	4	0
Japanese	4	0	3	1
Southeast Asian	3	0	2	1
Pacific	4	3	1	0
South American	7	4	2	1
Mexican	8	4	4	0
Mexican-American	9	2	4	3
Puerto Rican	8	1	2	5
CEPH-Utah	22	12	10	0
Unidentified	1	0	1	0
Total	119	43	53	23

a Frequency of the ancestral (=) sequence = 0.5840.

b Frequency of deletion (^*^552–^*^573del) = 0.4160.

**Figure 2:**
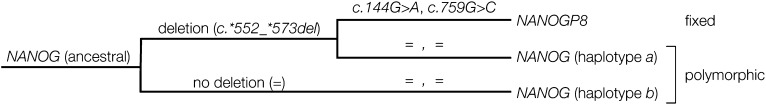
The evolutionary relationship of *NANOGP8* with the two major haplotypes of *NANOG*, as distinguished by evidently fixed reading-frame variants *c.144G > A* and *c.759G > C*, and the 22-nucleotide 3′ UTR deletion c.*552_*573del.

We found strong evidence in sequences we obtained and in genomic, EST, and SNP databases of two highly prevalent haplotypes of *NANOG* with worldwide distribution, as well as two intragenic recombinant haplotypes in a limited number of individuals, as detailed in File S3 and File S4. These two haplotypes differ by five substitution variants in the reading frame and by the *552–*573del deletion. This evidence also suggests that divergence of these two haplotypes predates the origin of *NANOGP8*. The haplotype derived from the parent allele of *NANOGP8* carries the derived deletion *552–*573del and is in the current primary human genome assembly. The other haplotype is in the current alternate genome assembly. Figure 2 summarizes the evolutionary relationships of *NANOGP8* with these two haplotypes of *NANOG*, portraying the relative times of occurrence for the two principal variants that are evidently fixed in *NANOGP8* (*c.144G > A* and *c.759G > C*), and deletion *552–*573del in *NANOGP8* and its parent allele. In Figure 2, we have named the two haplotypes in the primary and alternate assemblies, respectively, *a* and *b* to coincide with their designations as alleles *a* and *b* by Zbinden *et al.* (2010).

The presence of modern polymorphic variants in *NANOGP8* and between and within the two major haplotypes of *NANOG* has important implications for research on *NANOGP8* expression in cancer cells. All haplotypes of *NANOG* can be readily distinguished from *NANOGP8* in genomic DNA by the presence of introns in *NANOG* and the absence of introns in *NANOGP8*. Also, the 5′ insertion boundary of *NANOGP8* reliably distinguishes it from *NANOG*. We utilized these features to unambiguously distinguish *NANOG* and *NANOGP8* in our experiments. However, because RT-PCR products from *NANOG* and *NANOGP8* are derived from mRNAs, they lack these distinguishing features. Several published studies have relied on variants between *NANOG* and *NANOGP8* in reference sequences for primer design or for RFLPs to distinguish their RT-PCR products in cancer cells (Zhang *et al.* 2006; Jeter *et al.* 2009, 2011; Ambady *et al.* 2010; Zbinden *et al.* 2010; Eberle *et al.* 2010; Zhang *et al.* 2010; Ma *et al.* 2011, 2012; Uchino *et al.* 2012; Ibrahim *et al.* 2012). However, some of these variants, according to our data, are modern polymorphisms and, therefore, are unreliable for making these distinctions. Based on a detailed comparison of our data with data from these studies, as described in File S5, we determined that there is sufficient collective evidence from reliable fixed variants in sequenced RT-PCR products to confirm that *NANOGP8* is expressed in several different types of cancer cells and that its protein is the predominant form of NANOG in these cancer cells (Jeter *et al.* 2009, 2011; Ibrahim *et al.* 2012). However, some distinctions in previous studies of *NANOG* and *NANOGP8* RT-PCR products, and some conclusions regarding the presence or absence of *NANOGP8* expression in cancer cells, were based on unreliable polymorphic variants and may have been incorrect.

### *NANOGP8* is present in the Neanderthal genome

We also examined the Neanderthal genome in the region on chromosome 15 orthologous to the insertion site in the human genome of *NANOGP8* to determine whether *NANOGP8* is present in that genome. (By way of information, annotation of the current Neanderthal assembly is incorrect for *NANOG* and *NANOGP8*: *NANOG* on chromosome 12 is misidentified as *NANOGP8*, and *NANOGP8* on chromosome 15 is not identified.) Neanderthal sequences consist of short-read sequences aligned to the human reference assembly on the basis of sequence similarity. Because of the extremely high similarity of *NANOG* and *NANOGP8*, short-read sequences assigned to *NANOGP8* in the Neanderthal genome assembly may in fact be derived from *NANOG* or another *NANOG* pseudogene, or vice versa. Therefore, we searched for Neanderthal short-read sequences spanning sites that are unique to *NANOGP8* and capable of distinguishing it from the *NANOG* parent gene and other *NANOG* pseudogenes. The most reliable and unambiguous of such sites is the *NANOGP8* 5′-insertion boundary, which is unique to *NANOGP8*. We found two relatively long Neanderthal reads spanning this 5′-insertion boundary, one 78 and the other 57 nucleotides in length. We used these reads as queries for BLAST searches of the human genome and found no sites other than this insertion boundary in the human genome with significant similarity to the full lengths of each of these reads. Therefore, these two reads alone provide conclusive evidence that *NANOGP8* is present in the Neanderthal genome.

Also informative as confirmatory evidence are the exon splice sites in *NANOGP8*, which differ from *NANOG* and *NANOGP1* by their absence of introns, but may be similar to the paralogous splice sites in other *NANOG* retropseudogenes. A single read of 36 nucleotides spanning the splice site between exons 1 and 2 is assigned to *NANOGP8* in the Neanderthal genome assembly. However, this read has equal similarity to the paralogous site in *NANOGP9* and thus cannot be conclusively assigned to *NANOGP8*. A single read of 37 nucleotides spanning the splice site between exons 3 and 4 is identical only to the sequence at this site in *NANOGP8*. It differs by a single nucleotide from the paralogous site in *NANOGP7* and by two nucleotides from this site in *NANOGP4*. One of the derived variants in *NANOG* and *NANOGP8* that differs from *NANOGP7* and *NANOGP4* is a synonymous substitution variant (*c.498T > C*) that mutated in the parent *NANOG* gene after the origin of *NANOGP7* and *NANOGP4* (the most recent *NANOG* pseudogene before *NANOGP8*), prior to the hominin-panin divergence. In the human genome, it is present only in *NANOG*, *NANOGP1*, and *NANOGP8*, and the Neanderthal read containing it is distinguishable from *NANOG* and *NANOGP1* because it lacks the intron at the splice site between exons 3 and 4 in *NANOG* and *NANOGP1*. Therefore, this read is correctly assigned to *NANOGP8* in the Neanderthal genome.

The *c.759G > C p.Glu253His* substitution variant in *NANOGP8* (which, according to the sequences we obtained, is unique to and evidently fixed in *NANOGP8*) is present in all three reads assigned to this region in the Neanderthal genome. Therefore, these reads are correctly assigned to *NANOGP8*, and offer evidence that this mutation predates divergence the Neanderthal and modern-human ancestral lineages.

In summary, the presence of two reads spanning the 5′-insertion boundary of *NANOGP8*, the presence of a *NANOGP8*-specific read spanning the splice site between exons 3 and 4, and the presence of three reads with the *NANOGP8*-specific *c.759G > C p.Glu253His* substitution variant collectively offer definitive evidence that *NANOGP8* is present in the Neanderthal genome.

### *NANOGP8* is embedded in an SVA retroelement that may promote its transcription in cancer cells

SVA (SINE-R–VNTR–*Alu*) elements constitute a family of related composite retroelements composed of five segments: (1) a CCCCTC hexamer repeat on the 5′ end; (2) a segment composed of two truncated *Alu* elements in reverse orientation relative to the rest of the element; (3) a variable tandem nucleotide repeat (VNTR) segment; (4) a SINE-R segment derived from human endogenous retrovirus-K10 (HERV-K10), consisting of portions of the HERV-K10 *env* gene and the long terminal repeat (LTR); and (5) a poly(A) tail on the 3′ end of the element (Wang *et al.* 2005). They constitute the youngest class of retroelements in humans, having evolved about 13.5 million years ago exclusively in the human-great ape ancestral lineage shortly before divergence of the orangutan ancestral lineage from the human-chimpanzee-gorilla common ancestral lineage (Wang *et al.* 2005). Consequently, SVA elements are present exclusively in the genomes of humans and great apes. In the human genome, these elements fall into six subfamilies, named SVA_A through SVA_F. The SVA_A family, the most ancient, is found in the human, chimpanzee, gorilla, and orangutan genomes; it is retrotranspositionally quiescent in modern humans and great apes (Wang *et al.* 2005).

**Figure 3:**
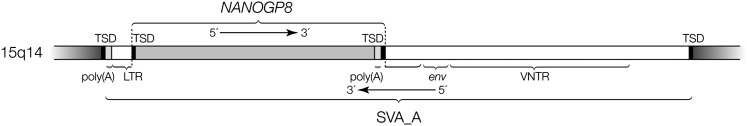
Insertion of *NANOGP8* into the LTR region of an SVA_A retroelement. env, *envelope* gene of a HERV-K endogenous retrovirus; LTR, long terminal repeat of a HERV-K endogenous retrovirus; poly(A), poly(A) tail; TSD, target site duplication; VNTR, variable nucleotide tandem repeat region.

We aligned the genomic region of human chromosome 15 consisting of *NANOGP8* plus 4000 nucleotides of flanking DNA sequence on both ends with orthologous sequences in the most recent genomic assemblies of common chimpanzee, Sumatran orangutan, and rhesus macaque. We also aligned two chromosome 15 contigs from gorilla whole-genome shotgun (WGS) sequences with portions of this region (gi 269664462 and gi 269664460). These alignments showed that *NANOGP8* is embedded in reverse orientation in the LTR region of an SVA_A retroelement in the human genome (Figure 3). This SVA_A retroelement is present in the human, chimpanzee, and gorilla genomes, but it is absent at the orthologous site in the orangutan and rhesus macaque genomes, indicating that it was inserted into chromosome 15 in the common ancestral lineage of humans, chimpanzees, and gorillas after divergence of the orangutan ancestral lineage. The evolutionary history of *NANOGP8* and its genomic context in the human genome, therefore, consists of insertion of an SVA_A element into the chromosome 15 homeolog in the human-chimpanzee-gorilla common ancestral lineage during a period between 8 and 16 million years ago (Locke *et al.* 2011), followed by insertion of *NANOGP8* into this SVA element exclusively in the human ancestral lineage approximately 0.9 to 2.5 million years ago, according to our estimate.

The core promoter elements of the LTR region of this SVA_A element reside in a 215 nucleotide-pair segment upstream of the 5′ border of *NANOGP8*, albeit in reverse orientation relative to *NANOGP8*. LTRs are known to possess promoter and enhancer activities in germline and cancer cells, although little is known about the specific promoter activity of SVA LTRs. Wang *et al.* (2005) suggested that SVA-LTR promoter elements may regulate transcription of genes residing near them and that transcription of SVA elements may extend beyond their borders as evidenced by transduced segments of flanking DNA carried by approximately 10% of retrotransposed SVA elements in the human genome.

The LTR in the common ancestor of all SVA elements was evolutionarily derived from a HERV-K LTR sequence, and the promoter activity of HERV-K LTRs has been extensively documented through experimentation (Fuchs *et al.* 2011). The LTR region upstream of the *NANOGP8* 5′ border has no recognizable TATA box and is in reverse orientation relative to *NANOGP8*. However, three experimental observations of HERV-K LTR promoters suggest that this SVA_A LTR may be capable of promoting transcription of *NANOGP8* in cancer cells. First, HERV-K genes are transcriptionally repressed in somatic cells but may gain transcriptional activity in germline and cancer cells (Cohen *et al.* 2009; Fuchs *et al.* 2011). Second, although the SVA_A LTR is in reverse orientation relative to *NANOGP8*, HERV-K promoters are capable of promoting antisense and bidirectional transcription (Dunn *et al.* 2006, Gogvadze *et al.* 2009). Third, HERV-K LTRs do not rely on canonical promoter sequences (such as the TATA box) but rather on transcription-factor binding sites, specifically Sp1 and Sp3 binding sites, shown experimentally to promote transcription in HERV-K LTRs (Fuchs *et al.* 2011).

We aligned the SVA LTR region upstream of *NANOGP8* with the HERV-K LTR core promoter sequences identified by Fuchs *et al.* (2011) and found that an Sp1/Sp3 binding site, named GC-box3, shown experimentally to be essential and functional as a transcription activator in a HERV-K LTR (see Figure 4 in Fuchs *et al.* 2011), is fully conserved at nucleotides –396 through –401 relative to the initiation codon of *NANOGP8*, albeit in reverse orientation. Moreover, computational analysis of the SVA LTR with TESS (Schug 2003) identified a potential Sp1 binding site at nucleotides –255 through –264 relative to the initiation codon, also in reverse orientation. Thus, the presence of inferred transcription-factor binding sites in this SVA_A LTR sequence and the position of *NANOGP8* relative to this sequence suggest a potential role for this LTR in promoting *NANOGP8*’s transcription in cancer cells. Transcription regulation may also extend to sequences upstream of the insertion border of the SVA element. Computational analysis with TFSEARCH (Heinemeyer *et al.* 1998) identified 95 potential transcription-factor binding sites within 1000 nucleotide pairs upstream of the *NANOGP8* initiation codon. This evidence is entirely inferential; the actual promoter elements and transcription factor binding sites that influence *NANOGP8* transcriptional activity are best determined experimentally, and the appropriate experiments are underway (Jeter *et al.* 2011).

## Conclusions

The principal conclusions of our research are as follows: *NANOGP8* is a human-specific retro-oncogene that arose approximately 0.9 to 2.5 million years ago in a common ancestor of humans and Neanderthals. Our evidence strongly indicates that it is fixed in modern humans, whereas its *NANOG* parent allele containing a derived deletion was polymorphic at the time of its origin and has remained polymorphic in one of two major haplotypes for *NANOG* in modern humans. The endogenous retroviral promoter elements present in an SVA LTR may promote transcription of *NANOGP8* in cancer cells, although current evidence supporting this proposition is inferential rather than experimental. The SVA_A element that carries *NANOGP8* in humans originated in a common ancestor of humans, chimpanzees, and gorillas after the divergence of the orangutan ancestral lineage.

Our observations of modern polymorphisms in *NANOG* and *NANOGP8* underscore the unreliability of variants between reference sequences for accurate experimental distinction of *NANOGP8* from *NANOG* RT-PCR products, particularly in studies of gene expression in cancer cells. Our observations further indicate that adequate screening in genetically diverse populations is essential to confirm which variants are modern polymorphisms and which are evidently fixed. Such screening is especially valuable if the variant may play a functional role in the protein or in gene expression. Furthermore, short-read sequences may be unreliable for polymorphism screening due to a high likelihood of incorrect assignment when a pseudogene or retrogene is highly similar to its parent gene, as *NANOGP8* is to *NANOG*.

The *c.759G > C p.Glu253His* variant in *NANOGP8* is of particular importance because it encodes the only fixed difference between the NANOG and NANOGP8 proteins. The mutant amino acid residue lies outside of the homeodomain, and its direct effect on the NANOGP8 protein’s biochemical function is unknown. Jeter *et al.* (2011) reported that most biological activities of the NANOG and NANOGP8 proteins are similar in cancer cells. However, their observations also suggest some enhanced tumorigenic functions for the NANOGP8 protein compared with the NANOG protein, possibly a consequence of this single amino acid substitution.

The major functional difference between *NANOG* and *NANOGP8* is at the level of gene expression. *NANOG* is expressed predominantly in embryonic stem cells where *NANOGP8* is apparently quiescent. By contrast, as Jeter *et al.* (2011) pointed out, “NANOGP8 is the predominant ‘isoform’ [of the protein] expressed in cancer cells and may, therefore, have evolved new functions distinct from those of NANOG1 in ESCs [embryonic stem cells]” (p. 11).

The recent evolution of *NANOGP8* as a human-specific retro-oncogene is highly relevant for cancer research because *NANOGP8* is absent in non-human species used as models for cancer. This situation may be a contributing factor to the delay in discovery of *NANOGP8* as a retro-oncogene, and it highlights the value of evolutionary and comparative genomic research to identify other potential oncogenes that may be lineage-specific to humans or have human-specific effects. Finally, the recent evolution of *NANOGP8* exclusively in the human ancestral lineage may partially explain some uniquely human aspects of cancer, including the higher predisposition for cancer in humans compared with other primates (Puente *et al.* 2006).

## Supplementary Material

Supporting Information
