# Supplementary material for: NANOGP8: Evolution of a Human-Specific Retro-Oncogene
Source: G3 (Bethesda). 2012 Nov 1;2(11):1447–57. doi: 10.1534/g3.112.004366 (PMC3484675; doi:10.1534/g3.112.004366)
Supplement: Supporting Information [file supp_2_11_1447__index.html]

Supporting Information 

# *NANOGP8*: Evolution of a Human-Specific Retro-Oncogene

## Supporting Information for Fairbanks *et al.*, 2012

**Files in this Data Supplement:**

- Supporting Information - Files S1-S5 (PDF, 175 KB)
- File S1 - Genotypes at ten single-nucleotide substitution variant positions in *NANOGP8* from cloned DNA obtained from 10 geographically diverse individuals (PDF, 80 KB)
- File S2 - Genotypes at six variant positions in *NANOGP8* from single-pass sequences in exon 4 from 94 geographically diverse individuals (PDF, 97 KB)
- File S3 - Genotypes at two single-nucleotide substitution variant positions in *NANOG* from single-pass sequences in exon 4 from 94 geographically diverse individuals, and genotypes for the 22 bp deletion at position \*552�\*573, as determined by PCR analysis in 119 geographically diverse individuals(PDF, 96 KB)
- File S4 - Evidence of Two Major Haplotypes of NANOG Throughout Human Populations Worldwide, and Intragenic Recombination Between Haplotypes (PDF, 87 KB)
- File S5 - An Analysis of Previously Published Efforts to Distinguish RT-PCR Products Derived from *NANOG* and *NANOGP8* in Cancer Cells (PDF, 93 KB)
