# Supplementary material for: NANOGP8: Evolution of a Human-Specific Retro-Oncogene
Source: G3 (Bethesda). 2012 Nov 1;2(11):1447–57. doi: 10.1534/g3.112.004366 (PMC3484675; doi:10.1534/g3.112.004366)
Supplement: Supporting Information [file supp_2.11.1447_004366SI.pdf]

File S1

Table S1 Genotypes at ten single-nucleotide substitution variant positions in *NANOGP8* from cloned DNA obtained from 10 geographically diverse individuals. For all but one individual (NA07038*D2), two clones were sequenced; the nucleotide in clone 1 is listed first, and the nucleotide in clone 2 listed second. Variants with a frequency of zero are variants identified in previous studies but not present in the sequences we examined.

**Coriell ID for DNA**	**Population**	**Nucleotide at Variant Positions in Coding Sequence** –135	**47**	**190**	**552**	**629**	**754**	***7**	***44**	***313**	***315**
NA17347*3	AFRICANS SOUTH OF THE SAHARA	T T	C C	G G	A A	C C	A A	G G	G G	C C	T T
NA17344*A1	AFRICANS SOUTH OF THE SAHARA	T T	C C	G G	A A	T C	A A	G G	G G	C C	T T
NA10472*A2	BIAKA PYGMY POPULATION	T C	C C	G G	A A	T C	A A	A G	G G	C C	C C
NA07038*D2	CEPH/UTAH PEDIGREE 1333	T	C	G	A	C	A	G	G	C	C
NA11521*3	DRUZE POPULATION	T T	C A	G G	A A	T C	A A	A G	G G	C C	C C
NA17030*1	INDO PAKISTANI	T T	C C	T T	G G	C C	A A	G G	G G	C C	C C
NA10492*A2	MBUTI PYGMY POPULATION	T T	C C	G G	A A	C C	A A	G G	A G	C C	C T
NA10496*A3	MBUTI PYGMY POPULATION	T T	C C	G G	A A	C C	A A	G G	G G	C C	T T
NA17387*1	PACIFIC	C T	C C	G T	A G	C C	A A	G G	G G	C C	C C
NA13618*2	RUSSIAN - KRASNODAR	T T	C C	G G	A A	C C	A A	G G	G G	C C	T T
Frequency of Derived Nucleotide		0.11	0.05	0.16	0.16	0.16	0.00	0.11	0.05	0.00	0.47

File S2

Table S2 Genotypes at six variant positions in *NANOGP8* from single-pass sequences in exon 4 from 94 geographically diverse individuals

Coriell ID for DNA Sample	Population	Genotype at Variant Positions in Coding Sequence					
		552	629	754	916–917	*7	*44
NA17347*3	AFRICANS SOUTH OF THE SAHARA	hom A/A	hom C/C	hom A/A	hom TG/TG	hom G/G	hom G/G
NA17344*A1	AFRICANS SOUTH OF THE SAHARA	hom A/A	het C/T	hom A/A	hom TG/TG	hom A/A	hom G/G
NA10472*A2	BIAKA PYGMY POPULATION	hom A/A	het C/T	hom A/A	hom TG/TG	het G/A	hom G/G
NA07038*D2	CEPH/UTAH PEDIGREE 1333	hom A/A	hom C/C	het A/C	het TG/del	hom G/G	
NA10492*A2	MBUTI PYGMY POPULATION	hom A/A	hom C/C	hom A/A	hom TG/TG	hom G/G	het G/A
NA10496*A3	MBUTI PYGMY POPULATION	hom A/A	hom C/C	hom A/A	hom TG/TG	hom G/G	hom G/G
NA17387*1	PACIFIC	het A/G	hom C/C	hom A/A	hom TG/TG	hom G/G	hom G/G
NA13618*2	RUSSIAN - KRASNODAR	hom A/A	hom C/C	hom A/A	hom TG/TG	hom G/G	hom G/G
NA17348*1	AFRICANS SOUTH OF THE SAHARA	hom A/A	hom C/C	hom A/A	hom TG/TG	hom G/G	het G/A
NA17343*1	AFRICANS SOUTH OF THE SAHARA	hom A/A	hom C/C	hom A/A	hom TG/TG	hom G/G	het G/A
NA10473*A3	BIAKA PYGMY POPULATION	hom A/A	hom C/C	hom A/A	hom TG/TG	hom G/G	het G/A
NA17033*1	AFRICAN AMERICAN	hom A/A	hom C/C	hom A/A	hom TG/TG	hom G/G	hom A/A
NA17172*A1	AFRICAN AMERICAN	hom A/A	het C/T	het A/C	het TG/del	het G/A	
NA17161*A2	AFRICAN AMERICAN	hom A/A	het C/T	hom A/A	hom TG/TG	het G/A	hom G/G
NA17062*A1	MEXICAN	hom A/A	het C/T	hom A/A	hom TG/TG	het G/A	hom G/G
NA17036*A1	AFRICAN AMERICAN	hom A/A	het C/T	hom A/A	hom TG/TG	hom A/A	hom G/G
NA13617*1	RUSSIAN - KRASNODA	hom A/A	het C/T	hom A/A	hom TG/TG	hom A/A	hom G/G
NA17032*A1	AFRICAN AMERICAN	hom A/A	het C/T	hom A/A	hom TG/TG	hom A/A	hom G/G

NA10470*A3	BIAKA PYGMY POPULATION	hom A/A	het C/T	hom A/A	hom TG/TG	hom A/A	hom G/G
NA17342*2	AFRICANS SOUTH OF THE SAHARA	hom A/A	hom C/C	hom A/A	hom TG/TG	hom A/A	hom G/G
NA13609*1	AMI POPULATION	hom A/A	hom T/T	hom A/A	hom TG/TG	hom A/A	hom G/G
NA17636*1	MEXICAN-AMERICAN	hom A/A	hom T/T	hom A/A	hom TG/TG	hom A/A	hom G/G
NA17341*2	AFRICANS SOUTH OF THE SAHARA	hom A/A	hom T/T	hom A/A	hom TG/TG	hom A/A	hom G/G
NA17057*4	JAPANESE	hom A/A	hom T/T	hom A/A	hom TG/TG	hom A/A	hom G/G
NA17028*2	INDO PAKISTANI	het A/G	hom C/C	hom A/A	hom TG/TG	hom G/G	hom G/G
NA17317*A1	SOUTH AMERICA	het A/G	hom C/C	hom A/A	hom TG/TG	hom G/G	hom G/G
NA17076*2	PUERTO RICAN	het A/G	hom C/C	hom A/A	hom TG/TG	hom G/G	hom G/G
NA17065*A5	MEXICAN	het A/G	hom C/C	hom A/A	hom TG/TG	hom G/G	hom G/G
NA10849*5	CEPH/UTAH PEDIGREE 1332	het A/G	hom C/C	hom A/A	hom TG/TG	hom G/G	hom G/G
NA12911*2	CEPH/UTAH PEDIGREE 1582	het A/G	hom C/C	hom A/A	hom TG/TG	hom G/G	hom G/G
NA17313*A1	SOUTH AMERICA	het A/G	hom C/C	hom A/A	hom TG/TG	hom G/G	hom G/G
NA17040*A1	AFRICAN AMERICAN	hom A/A	hom C/C	hom A/A	hom TG/TG	hom G/G	hom G/G
NA17039*1	AFRICAN AMERICAN	hom A/A	hom C/C	hom A/A	hom TG/TG	hom G/G	hom G/G
NA17037*1	AFRICAN AMERICAN	hom A/A	hom C/C	hom A/A	hom TG/TG	hom G/G	hom G/G
NA17166*A1	AFRICAN AMERICAN	hom A/A	hom C/C	hom A/A	hom TG/TG	hom G/G	hom G/G
NA17345*2	AFRICANS SOUTH OF THE SAHARA	hom A/A	hom C/C	hom A/A	hom TG/TG	hom G/G	hom G/G
NA17346*2	AFRICANS SOUTH OF THE SAHARA	hom A/A	hom C/C	hom A/A	hom TG/TG	hom G/G	hom G/G
NA07057*D2	CEPH/UTAH PEDIGREE 1331	hom A/A	hom C/C	hom A/A	hom TG/TG	hom G/G	hom G/G
NA10858*B1	CEPH/UTAH PEDIGREE 1347	hom A/A	hom C/C	hom A/A	hom TG/TG	hom G/G	hom G/G
NA11993*C1	CEPH/UTAH PEDIGREE 1362	hom A/A	hom C/C	hom A/A	hom TG/TG	hom G/G	hom G/G
NA12909*2	CEPH/UTAH PEDIGREE 1477	hom A/A	hom C/C	hom A/A	hom TG/TG	hom G/G	hom G/G
NA17710*1	MEXICAN-AMERICAN	hom A/A	hom C/C	hom A/A	hom TG/TG	hom G/G	hom G/G
NA17443*2	MEXICAN-AMERICAN	hom A/A	hom C/C	hom A/A	hom TG/TG	hom G/G	hom G/G
NA18460*1	NOT IDENTIFIED	hom A/A	hom C/C	hom A/A	hom TG/TG	hom G/G	hom G/G

NA17072*A1	PUERTO RICAN	hom A/A	hom C/C	hom A/A	hom TG/TG	hom G/G	hom G/G
NA17071*A2	PUERTO RICAN	hom A/A	hom C/C	hom A/A	hom TG/TG	hom G/G	hom G/G
NA17314*2	SOUTH AMERICA	hom A/A	hom C/C	hom A/A	hom TG/TG	hom G/G	hom G/G
NA17088*2	SOUTHEAST ASIANS	hom A/A	hom C/C	hom A/A	hom TG/TG	hom G/G	hom G/G
NA12273*B2	CEPH/UTAH PEDIGREE 1418	hom A/A	hom C/C	hom A/A	hom TG/TG	hom G/G	hom G/G
NA11522*4	DRUZE POPULATION	hom A/A	hom C/C	hom A/A	hom TG/TG	hom G/G	hom G/G
NA11524*3	DRUZE POPULATION	hom A/A	hom C/C	hom A/A	hom TG/TG	hom G/G	hom G/G
NA17066*2	MEXICAN	hom A/A	hom C/C	hom A/A	hom TG/TG	hom G/G	hom G/G
NA17634*1	MEXICAN-AMERICAN	hom A/A	hom C/C	hom A/A	hom TG/TG	hom G/G	hom G/G
NA17311*A5	SOUTH AMERICA	hom A/A	hom C/C	hom A/A	hom TG/TG	hom G/G	hom G/G
NA06990*F1	CEPH/UTAH PEDIGREE 1331	hom A/A	hom C/C	hom A/A	hom TG/TG	hom G/G	hom G/G
NA17017*5	CHINESE (VERSION 1)	hom A/A	hom C/C	hom A/A	hom TG/TG	hom G/G	hom G/G
NA17016*3	CHINESE (VERSION 1)	hom A/A	hom C/C	hom A/A	hom TG/TG	hom G/G	hom G/G
NA17058*3	JAPANESE	hom A/A	hom C/C	hom A/A	hom TG/TG	hom G/G	hom G/G
NA17060*3	JAPANESE	hom A/A	hom C/C	hom A/A	hom TG/TG	hom G/G	hom G/G
NA17391*2	PACIFIC	hom A/A	hom C/C	hom A/A	hom TG/TG	hom G/G	hom G/G
NA10832*3	CEPH/UTAH PEDIGREE 1413	hom A/A	hom C/C	hom A/A	hom TG/TG	hom G/G	hom G/G
NA17389*2	PACIFIC	hom A/A	hom C/C	hom A/A	hom TG/TG	hom G/G	hom G/G
NA17388*1	PACIFIC	hom A/A	hom C/C	hom A/A	hom TG/TG	hom G/G	hom G/G
NA17073*A1	PUERTO RICAN	hom A/A	hom C/C	hom A/A	hom TG/TG	hom G/G	hom G/G
NA17056*B2	JAPANESE	hom A/A	hom C/C	hom A/A	hom TG/TG	hom G/G	hom G/G
NA10494*A3	MBUTI PYGMY POPULATION	hom A/A	hom C/C	hom A/A	hom TG/TG	hom G/G	hom G/G
NA17700*1	MEXICAN-AMERICAN	hom A/A	hom C/C	hom A/A	hom TG/TG	hom G/G	hom G/G
NA17075*2	PUERTO RICAN	hom A/A	hom C/C	hom A/A	hom TG/TG	hom G/G	hom G/G
NA17074*3	PUERTO RICAN	hom A/A	hom C/C	hom A/A	hom TG/TG	hom G/G	hom G/G
NA17315*1	SOUTH AMERICA	hom A/A	hom C/C	hom A/A	hom TG/TG	hom G/G	hom G/G

NA17087*2	SOUTHEAST ASIANS	hom A/A	hom C/C	hom A/A	hom TG/TG	hom G/G	hom G/G
NA17316*2	SOUTH AMERICA	hom A/A	hom C/C	hom A/A	hom TG/TG	hom G/G	hom G/G
NA17167*2	AFRICAN AMERICAN	hom A/A	hom C/C	hom A/A	hom TG/TG	hom G/G	hom G/G
NA07349*B1	CEPH/UTAH PEDIGREE 1345	hom A/A	hom C/C	hom A/A	hom TG/TG	hom G/G	hom G/G
NA10860*B1	CEPH/UTAH PEDIGREE 1362	hom A/A	hom C/C	hom A/A	hom TG/TG	hom G/G	hom G/G
NA10831*A7	CEPH/UTAH PEDIGREE 1408	hom A/A	hom C/C	hom A/A	hom TG/TG	hom G/G	hom G/G
NA10833*4	CEPH/UTAH PEDIGREE 1413	hom A/A	hom C/C	hom A/A	hom TG/TG	hom G/G	hom G/G
NA12813*5	CEPH/UTAH PEDIGREE 1454	hom A/A	hom C/C	hom A/A	hom TG/TG	hom G/G	hom G/G
NA12841*3	CEPH/UTAH PEDIGREE 1458	hom A/A	hom C/C	hom A/A	hom TG/TG	hom G/G	hom G/G
NA11523*3	DRUZE POPULATION	hom A/A	hom C/C	hom A/A	hom TG/TG	hom G/G	hom G/G
NA11525*3	DRUZE POPULATION	hom A/A	hom C/C	hom A/A	hom TG/TG	hom G/G	hom G/G
NA17067*A1	MEXICAN	hom A/A	hom C/C	hom A/A	hom TG/TG	hom G/G	hom G/G
NA17701*2	MEXICAN-AMERICAN	hom A/A	hom C/C	hom A/A	hom TG/TG	hom G/G	hom G/G
NA13611*2	AMI POPULATION	hom G/G	hom C/C	hom A/A	hom TG/TG	hom G/G	hom G/G
NA13607*5	AMI POPULATION	hom G/G	hom C/C	hom A/A	hom TG/TG	hom G/G	hom G/G
NA17349*3	AFRICANS SOUTH OF THE SAHARA	hom A/A	hom T/T	hom A/A	hom TG/TG	hom G/G	hom G/G
NA06987*D6	CEPH/UTAH PEDIGREE 1333	hom A/A	hom C/C	hom C/C	hom del	hom G/G	hom G/G
NA17158*3	AFRICAN AMERICAN	hom A/A	hom C/C	hom C/C	hom del	hom G/G	hom G/G
NA17035*A1	AFRICAN AMERICAN	hom A/A	het C/T	het A/C	het TG/del	het G/A	
NA17038*A1	AFRICAN AMERICAN	hom A/A	het C/T	het A/C	het TG/del	het G/A	
NA17061*A3	MEXICAN	hom A/A	hom C/C	het A/C	het TG/del	hom G/G	
NA17034*A2	AFRICAN AMERICAN	hom A/A	hom C/C	het A/C	het TG/del	hom G/G	
NA17078*1	PUERTO RICAN	hom A/A	hom C/C	het A/C	het TG/del	hom G/G	
NA10861*B3	CEPH/UTAH PEDIGREE 1362	het A/G	hom C/C	hom A/A	het TG/del	hom G/G	
Frequency of Derived Nucleotide		0.07	0.11	0.06	0.06	0.14	0.03

File S3

Table S3 Genotypes at two single-nucleotide substitution variant positions in *NANOG* from single-pass sequences in exon 4 from 94 geographically diverse individuals, and genotypes for the 22 bp deletion at position *552–*573, as determined by PCR analysis in 119 geographically diverse individuals.

Coriell ID for DNA Sample	Population	Genotype at Variant Positions in Coding Sequence		
		531	798	*552–*573
NA17347*3	AFRICANS SOUTH OF THE SAHARA	het C/T	het C/T	het =/del
NA17344*A1	AFRICANS SOUTH OF THE SAHARA	hom C/C	het C/T	hom del/del
NA10472*A2	BIAKA PYGMY POPULATION	hom T/T	hom C/C	hom del/del
NA07038*D2	CEPH/UTAH PEDIGREE 1333	hom C/C	hom T/T	hom =/=
NA11521*3	DRUZE POPULATION			hom =/=
NA17030*1	INDO PAKISTANI			hom =/=
NA10492*A2	MBUTI PYGMY POPULATION	hom C/C	hom T/T	hom =/=
NA10496*A3	MBUTI PYGMY POPULATION	hom T/T	hom C/C	het =/del
NA17387*1	PACIFIC	hom T/T	hom C/C	hom =/=
NA13618*2	RUSSIAN - KRASNODAR	het C/T	het C/T	hom =/=
NA17348*1	AFRICANS SOUTH OF THE SAHARA	het C/T	het C/T	het =/del
NA17343*1	AFRICANS SOUTH OF THE SAHARA	het C/T	het C/T	het =/del
NA10473*A3	BIAKA PYGMY POPULATION	het C/T	het C/T	het =/del
NA17033*1	AFRICAN AMERICAN	het C/T	het C/T	het =/del
NA17172*A1	AFRICAN AMERICAN	het C/T	het C/T	het =/del
NA17161*A2	AFRICAN AMERICAN	hom T/T	hom C/C	hom del/del
NA17062*A1	MEXICAN	hom C/C	hom T/T	hom =/=

NA17036*A1	AFRICAN AMERICAN	het C/T	het C/T	het =/del
NA13617*1	RUSSIAN - KRASNODA	het C/T	het C/T	het =/del
NA17032*A1	AFRICAN AMERICAN	hom T/T	hom C/C	hom del/del
NA10470*A3	BIAKA PYGMY POPULATION	hom C/C	hom T/T	hom =/=
NA17342*2	AFRICANS SOUTH OF THE SAHARA	het C/T	het C/T	het =/del
NA13609*1	AMI POPULATION	het C/T	het C/T	het =/del
NA17636*1	MEXICAN-AMERICAN	het C/T	het C/T	het =/del
NA17341*2	AFRICANS SOUTH OF THE SAHARA	het C/T	het C/T	hom del/del
NA17057*4	JAPANESE	hom T/T	hom C/C	het =/del
NA17028*2	INDO PAKISTANI	het C/T	het C/T	hom =/=
NA17317*A1	SOUTH AMERICA	het C/T	het C/T	hom =/=
NA17076*2	PUERTO RICAN	het C/T	het C/T	hom del/del
NA17065*A5	MEXICAN	hom T/T	hom C/C	het =/del
NA10849*5	CEPH/UTAH PEDIGREE 1332	hom C/C	hom T/T	hom =/=
NA12911*2	CEPH/UTAH PEDIGREE 1582	hom C/C	hom T/T	hom =/=
NA17313*A1	SOUTH AMERICA	hom C/C	hom T/T	hom =/=
NA17040*A1	AFRICAN AMERICAN	het C/T	het C/T	het =/del
NA17039*1	AFRICAN AMERICAN	het C/T	het C/T	het =/del
NA17037*1	AFRICAN AMERICAN	het C/T	het C/T	het =/del
NA17166*A1	AFRICAN AMERICAN	het C/T	het C/T	het =/del
NA17345*2	AFRICANS SOUTH OF THE SAHARA	het C/T	het C/T	het =/del
NA17346*2	AFRICANS SOUTH OF THE SAHARA	het C/T	het C/T	het =/del
NA07057*D2	CEPH/UTAH PEDIGREE 1331	het C/T	het C/T	het =/del
NA10858*B1	CEPH/UTAH PEDIGREE 1347	het C/T	het C/T	het =/del
NA11993*C1	CEPH/UTAH PEDIGREE 1362	het C/T	het C/T	het =/del
NA12909*2	CEPH/UTAH PEDIGREE 1477	het C/T	het C/T	het =/del

NA17710*1	MEXICAN-AMERICAN	het C/T	het C/T	het =/del
NA17443*2	MEXICAN-AMERICAN	het C/T	het C/T	het =/del
NA18460*1	NOT IDENTIFIED	het C/T	het C/T	het =/del
NA17072*A1	PUERTO RICAN	het C/T	het C/T	het =/del
NA17071*A2	PUERTO RICAN	het C/T	het C/T	het =/del
NA17314*2	SOUTH AMERICA	het C/T	het C/T	het =/del
NA17088*2	SOUTHEAST ASIANS	het C/T	het C/T	het =/del
NA12273*B2	CEPH/UTAH PEDIGREE 1418	het C/T	het C/T	hom =/=
NA11522*4	DRUZE POPULATION	het C/T	het C/T	hom =/=
NA11524*3	DRUZE POPULATION	het C/T	het C/T	hom =/=
NA17066*2	MEXICAN	het C/T	het C/T	hom =/=
NA17634*1	MEXICAN-AMERICAN	het C/T	het C/T	hom =/=
NA17311*A5	SOUTH AMERICA	het C/T	het C/T	hom =/=
NA06990*F1	CEPH/UTAH PEDIGREE 1331	hom T/T	hom C/C	het =/del
NA17017*5	CHINESE (VERSION 1)	hom T/T	hom C/C	het =/del
NA17016*3	CHINESE (VERSION 1)	hom T/T	hom C/C	het =/del
NA17058*3	JAPANESE	hom T/T	hom C/C	het =/del
NA17060*3	JAPANESE	hom T/T	hom C/C	het =/del
NA17391*2	PACIFIC	hom T/T	hom C/C	het =/del
NA10832*3	CEPH/UTAH PEDIGREE 1413	hom T/T	hom C/C	hom =/=
NA17389*2	PACIFIC	hom T/T	hom C/C	hom =/=
NA17388*1	PACIFIC	hom T/T	hom C/C	hom =/=
NA17073*A1	PUERTO RICAN	hom T/T	hom C/C	hom =/=
NA17056*B2	JAPANESE	hom T/T	hom C/C	hom del/del
NA10494*A3	MBUTI PYGMY POPULATION	hom T/T	hom C/C	hom del/del
NA17700*1	MEXICAN-AMERICAN	hom T/T	hom C/C	hom del/del

NA17075*2	PUERTO RICAN	hom T/T	hom C/C	hom del/del
NA17074*3	PUERTO RICAN	hom T/T	hom C/C	hom del/del
NA17315*1	SOUTH AMERICA	hom T/T	hom C/C	hom del/del
NA17087*2	SOUTHEAST ASIANS	hom T/T	hom C/C	hom del/del
NA17316*2	SOUTH AMERICA	hom C/C	hom T/T	het =/del
NA17167*2	AFRICAN AMERICAN	hom C/C	hom T/T	hom =/=
NA07349*B1	CEPH/UTAH PEDIGREE 1345	hom C/C	hom T/T	hom =/=
NA10860*B1	CEPH/UTAH PEDIGREE 1362	hom C/C	hom T/T	hom =/=
NA10831*A7	CEPH/UTAH PEDIGREE 1408	hom C/C	hom T/T	hom =/=
NA10833*4	CEPH/UTAH PEDIGREE 1413	hom C/C	hom T/T	hom =/=
NA12813*5	CEPH/UTAH PEDIGREE 1454	hom C/C	hom T/T	hom =/=
NA12841*3	CEPH/UTAH PEDIGREE 1458	hom C/C	hom T/T	hom =/=
NA11523*3	DRUZE POPULATION	hom C/C	hom T/T	hom =/=
NA11525*3	DRUZE POPULATION	hom C/C	hom T/T	hom =/=
NA17067*A1	MEXICAN	hom C/C	hom T/T	hom =/=
NA17701*2	MEXICAN-AMERICAN	hom C/C	hom T/T	hom =/=
NA13611*2	AMI POPULATION	hom T/T	hom C/C	het =/del
NA13607*5	AMI POPULATION	hom T/T	hom C/C	hom del/del
NA17349*3	AFRICANS SOUTH OF THE SAHARA	hom C/C	hom T/T	hom =/=
NA06987*D6	CEPH/UTAH PEDIGREE 1333	het C/T	het C/T	het =/del
NA17158*3	AFRICAN AMERICAN	hom C/C	hom T/T	hom =/=
NA17035*A1	AFRICAN AMERICAN	het C/T	het C/T	hom del/del
NA17038*A1	AFRICAN AMERICAN	hom T/T	hom C/C	het =/del
NA17061*A3	MEXICAN	het C/T	het C/T	het =/del
NA17034*A2	AFRICAN AMERICAN	hom T/T	hom C/C	hom del/del
NA17078*1	PUERTO RICAN	hom T/T	hom C/C	hom del/del

NA10861*B3	CEPH/UTAH PEDIGREE 1362	het C/T	het C/T	het =/del
NA10469*B1	BIAKA PYGMY POPULATION			het =/del
NA10859*D3	CEPH/UTAH PEDIGREE 1347			het =/del
NA12749*3	CEPH/UTAH PEDIGREE 1444			het =/del
NA12912*3	CEPH/UTAH PEDIGREE 1582			het =/del
NA17018*A2	CHINESE			het =/del
NA17019*A2	CHINESE			het =/del
NA17063*2	MEXICAN			het =/del
NA17068*A2	MEXICAN			het =/del
NA17698*1	MEXICAN-AMERICAN			het =/del
NA17086*2	SOUTHEAST ASIANS			het =/del
NA07348*F1	CEPH/UTAH PEDIGREE 1345			hom =/=
NA17020*A1	CHINESE (VERSION 1)			hom =/=
NA17029*1	INDO PAKISTANI			hom =/=
NA10495*B1	MBUTI PYGMY POPULATION			hom =/=
NA17064*A2	MEXICAN			hom =/=
NA13619*2	RUSSIAN - KRASNODAR			hom =/=
NA17312*2	SOUTH AMERICA			hom =/=
NA13610*2	AMI POPULATION			hom del/del
NA13608*2	AMI POPULATION			hom del/del
NA10493*A1	MBUTI PYGMY POPULATION			hom del/del
NA17448*2	MEXICAN-AMERICAN			hom del/del
NA17684*1	MEXICAN-AMERICAN			hom del/del
NA17077*A1	PUERTO RICAN			hom del/del

File S4

Evidence of Two Major Haplotypes of *NANOG* Throughout Human Populations Worldwide, and Intragenic Recombination Between Haplotypes

Sequences we obtained of *NANOG* exon 4 from 94 geographically diverse individuals revealed two highly polymorphic synonymous substitution variants, *c.531C>T* and *c.798C>T*, in the reading frame. In both cases, C is ancestral and T derived. In *NANOGP8*, the ancestral nucleotides at both positions were homozygous in all 94 individuals, evidence that the parent allele of *NANOG* at the time of *NANOGP8*'s origin carried the ancestral nucleotides at both sites. We were able to distinguish homozygotes and heterozygotes for these two substitution polymorphisms in all 94 individuals tested, and compared them to the genotypes previously determined for the _*552–*573del_ deletion. As shown in Table S4, of the 27 possible genotypes for these three polymorphisms, only nine were observed.

Hereafter, and in Table S4, we denote haplotypes for these variants as abbreviations in accordance with current human haplotype nomenclature (http://www.hgvs.org/mutnomen) as follows: For positions c.531 and c.798, the nucleotide is listed (C or T). For position c.*552–*573, = denotes the ancestral non-deleted sequence, and *del* the derived deleted sequence. Semicolons separate variants at these three sites in their respective order.

Haplotypes could be conclusively identified only in triple homozygotes and individuals heterozygous for one of the three variants, a total of 51 individuals. The two most prevalent haplotypes are the reciprocal haplotypes *T*; *C*; *del* and *C*; *T*; =, which, respectively, are the two haplotypes in the current primary and alternate genomic assemblies of *NANOG*. The third most frequent haplotype is *T*; *C*; =, which probably arose through intragenic recombination between the two most prevalent haplotypes. Its reciprocal haplotype, *C*; *T*; *del*, also an apparent intragenic recombinant, is rare in the samples we examined, confirmed to be present in the heterozygous condition in five individuals. Only one individual (from the Africans South of the Sahara panel) carries as a heterozygote the fifth haplotype, *C*; *C*; *del*, which is the ancestral parent haplotype of *NANOGP8*. As shown in Table S4, double and triple heterozygotes could best be explained as genotypes heterozygous for the four most prevalent haplotypes; alternative genotypes for double and triple heterozygotes require the *T*; *T*; = or *T*; *T*; *del* haplotypes, which were not observed in any triple homozygotes or single heterozygotes.

Minor allele frequencies (MAFs) in the *NANOG* NCBI dbSNP report for five variants in the reading frame (*c.165C>T*, *c.246T>G*, *c.276G>A*, *c.531C>T*, and *c.798C>T*) are essentially equal, ranging from 0.3768 to 0.3863. In all five cases, the minor allele (two of which are ancestral and three derived) is the nucleotide present in the alternate

reference assembly, which corresponds to haplotype *C; T; =* in our sequences. Also, for each of these five variants, the major allele is the nucleotide present in the primary reference assembly (three ancestral and two derived), which corresponds to haplotype *T; C; del* in our sequences. These observations suggest that the *NANOG* sequences in the primary and alternate assemblies represent two major haplotypes that differ by five substitution variants within the reading frame. They correspond, respectively, to alleles *a* and *b* of *NANOG* identified by Zbiden *et al.* (2010). We used the reading-frame sequence of the primary and alternate assemblies of *NANOG* as a query for MEGABLAST searches (using default parameters) against the human EST database and identified 21 ESTs with 98% or greater similarity spanning at least two of these variant sites. Fifteen of these ESTs had a sequence consistent with the primary-assembly haplotype and six with the alternate-assembly haplotype, with no identifiable intragenic recombinants within the reading frame, further evidence of two prevalent haplotypes for *NANOG*.

Table S4 A. Observed genotypes and inferred haplotypes in *NANOG* for the *c.531C>T*, *c.798C>T*, and **552–*573del* derived variants and their ancestral counterparts (=).

**Observed Genotype**	**Number of Individuals**	**Inferred Haplotypes**
Triple homozygote *C/C; T/T; =/=*	20	*C; T; =*
Triple homozygote *T/T; C/C; del/del*	13	*T; C; del*
Triple homozygote *T/T; C/C; =/=*	5	*T; C; =*
Single heterozygote *T/T; C/C; =/del*	11	*T; C; =/T; C; del*
Single heterozygote *C/C; T/T; =/del*	1	*C; T; =/C; T; del*
Single heterozygote *C/C; C/T; del/del*	1	*C; T; del/C; C; del*
Double heterozygote *C/T; C/T; =/=*	9	*C; T; =/T; C; =* (most probable)
Double heterozygote *C/T; C/T; del/del*	3	*T; C; del/C; T; del* (most probable)
Triple heterozygote *C/T; C/T; =/del*	31	*C; T; =/ T; C; del* (most probable)
**Total**	**94**	

B. Number and frequency of chromosomes with inferred haplotypes

Haplotype	Number of Chromosomes	Frequency
C; T; = (major haplotype, alternate assembly)	81	0.4309
T; C; del (major haplotype, primary assembly)	71	0.3777
T; C; = (recombinant haplotype)	30	0.1596
C; T; del (recombinant haplotype)	5	0.0266
C; C; del (ancestral haplotype)	1	0.0053

File S5

An Analysis of Previously Published Efforts to Distinguish RT-PCR Products Derived from *NANOG* and *NANOGP8* in Cancer Cells

Because both *NANOG* and *NANOGP8* may be transcriptionally active in cancer cells, accurate distinction of their RT-PCR products is essential for gene-expression research. The genomic differences between *NANOG* and *NANOGP8* are considerably greater than those in RT-PCR products, due to the presence of introns in *NANOG* and their absence in *NANOGP8*, and different flanking sequences at the genomic borders of *NANOG* and *NANOGP8* the insertion boundaries of *NANOGP8*. Ultimately, RT-PCR products must be distinguished based on variants within the mRNA that differ between the two in the cell cultures under study. Researchers who have studied differential expression of *NANOG* and *NANOGP8* in cancer cells have relied on a variety of differences between *NANOG* and *NANOGP8* sequences in genomic reference assemblies for experimental distinction of RT-PCR products. According to our experimental results, however, differences in reference assemblies may be unreliable because they may represent modern polymorphisms present in a subset of individuals.

Zhang et al. (2006) published the first evidence that *NANOGP8* is a retrogene expressed in cancer cells. They utilized primers capable of amplifying RT-PCR products from both *NANOG* and *NANOGP8*, but distinguished the two through sequencing the reading frame of their RT-PCR products. The sequences they identified as belonging to *NANOGP8* contained variants we identified as evidently fixed in *NANOGP8* (*c.144G>A* and *c.759G>C*), confirming the accuracy of their identifications. Likewise, Zhang et al. (2010) and Uchino et al. (2012) utilized primers capable of amplifying RT-PCR products from both *NANOG* and *NANOGP8* and sequenced their RT-PCR products confirming their correct identities.

Jeter et al. (2009, 2011), Ma et al. (2010, 2012), and Ibrahim et al. (2012) relied on the 22-nucleotide pair deletion in the 3′ UTR of *NANOGP8* (c.*552_*573del) as a site for primers and probes to distinguish *NANOG* from *NANOGP8* qRT-PCR products, presuming this deletion to be unique to *NANOGP8* based on an earlier human genome reference assembly. Our results demonstrate that this deletion is uniformly present in *NANOGP8* but highly polymorphic in *NANOG*. Its use as a primer-binding site for RT-PCR should result in reliable amplification of *NANOGP8* fragments but may also result in amplification of fragments of identical size from *NANOG*, if *NANOG* is transcribed in those

cells and if the cells are from individuals who carry the *c.*552_*573del* allele of *NANOG* (a majority of individuals according to our results). Jeter et al. (2009) and Ibrahim *et al.* (2012) sequenced RT-PCR products they obtained, confirming the correct identifications of these products as belonging to *NANOGP8* or *NANOG* on the basis of reading-frame variants that, according to our research, are evidently fixed. There is no indication of sequencing to confirm correct identification of RT-PCR products in other studies utilizing *c.*552_*573del* as primer-binding site (Jeter et al. 2011, Ma et al. 2010, 2012).

Ambady et al. (2010) relied on a *Sma*I RFLP generated by a derived substitution variant in the 3´ UTR (*c*313C>G*) to distinguish *NANOG* and *NANOGP8* RT-PCR products, presuming the *Sma*I site to be unique to *NANOG* based on reference-sequence comparison. Our results, however, demonstrate that the ancestral *Sma*I site and the derived variant in *NANOGP8* that alters the site to create the RFLP are highly polymorphic in *NANOGP8*, rendering this RFLP unreliable for distinguishing *NANOG* and *NANOGP8* RT-PCR products. However, Ambady et al. (2010) sequenced the RT-PCR products they obtained, confirming their correct identity as *NANOGP8*.

Zbinden et al. (2010) sequenced a region they referred to as "a diagnostic 3´ UTR region, which varies among the *NANOG* alleles and *NANOGP8*" (p. 2660), based on their comparison of reference sequences. However, they did not specify which variants they considered as diagnostic. According to our results, all variants in the 3´ UTR are modern polymorphisms in either *NANOG* or *NANOGP8*, except **606T>G* in *NANOGP8*, which we could not confirm as fixed or polymorphic.

Eberle et al. (2010) utilized RT-PCR to detect *NANOG* transcripts in acute leukemic human cell lines, and concluded that *NANOGP8* was not expressed in these cells. Their conclusion was based on two primer pairs (which they named set a and set b) presumed to amplify fragments from transcripts of both *NANOG* and *NANOGP8*, as well as other *NANOG* pseudogenes. They sequenced the RT-PCR products from these primer pairs but did not state which variants they considered to be reliable identifiers of *NANOG* and *NANOGP8*. One of their primer pairs (set a) should have amplified fragments of identical size from both *NANOG* and *NANOGP8* transcripts, consisting of most of the reading frame. The reverse primer of the other pair (set b), however, had on its 3´ end the ancestral G at site c.759, which is present in *NANOG*, but altered in *NANOGP8* by the *c.759G>C* fixed variant. Therefore, this primer pair should have successfully amplified fragments from *NANOG* but not *NANOGP8*. To confirm their conclusion that *NANOGP8* was not expressed in these cells, they utilized a third primer pair they considered to be exclusive to *NANOGP8*, and detected no amplification in any of 60 clones from a single cell line. This primer pair, however, relied on the *c.47C>A* variant on the 3´ end of the forward primer for exclusive amplification of *NANOGP8*. Although this derived variant is present in both the current primary and alternate reference assemblies of *NANOGP8*, our results, as well as those of Jeter et al. (2009) and Uchino et al. (2012), show it to be polymorphic

and rare in *NANOGP8*. Moreover, the reverse primer in this pair had the derived T in the *c.531C>T* variant as the third nucleotide from the 3´ end of the primer, which is absent in *NANOGP8* and polymorphic in *NANOG*, according to our sequences. Therefore, this primer pair, considered to be *NANOGP8*-specific, is not likely to amplify fragments from either *NANOGP8* or *NANOG* in most individuals, and may explain the lack of amplification observed. Beyond these issues for *NANOGP8* identification, the results of Eberle et al. (2010) are highly pertinent in that alternative splicing of transcripts from *NANOG* and *NANOGP1* was well documented in these cell lines.

Ishiguro et al. (2012) relied on an RFLP resulting from the *c.144G>A* variant in *NANOGP8* to distinguish *NANOG* and *NANOGP8* RT-PCR products. Our data suggest that this is a reliable variant due to evident fixation of the ancestral allele in *NANOG* and the derived allele in *NANOGP8*.

Our observations of widespread modern polymorphisms in *NANOG* and *NANOGP8* underscore the unreliability of variants between reference sequences for accurate experimental identification of RT-PCR products. Instead, the most reliable method to distinguish *NANOG* from *NANOGP8* RT-PCR products is to sequence genomic-DNA specific to *NANOG* and *NANOGP8* from the cell lines being researched to identify which variants distinguish the two in any particular cell line, then use those variants to accurately identify sequenced RT-PCR products for each line. Several of the primer pairs we used (see Table 1 in the main text of the article) are capable of generating PCR fragments specific to *NANOG* or *NANOGP8* from genomic DNA (albeit not from mRNA for RT-PCR), and may be useful for such genomic-DNA sequencing.
