# Supplementary material for: NANOGP8: Evolution of a Human-Specific Retro-Oncogene
Source: G3 (Bethesda). 2012 Nov 1;2(11):1447–57. doi: 10.1534/g3.112.004366 (PMC3484675; doi:10.1534/g3.112.004366)
Supplement: Supporting Information [file supp_2.11.1447_FileS1.pdf]

File S1

Table S1 Genotypes at ten single-nucleotide substitution variant positions in *NANOGP8* from cloned DNA obtained from 10 geographically diverse individuals. For all but one individual (NA07038*D2), two clones were sequenced; the nucleotide in clone 1 is listed first, and the nucleotide in clone 2 listed second. Variants with a frequency of zero are variants identified in previous studies but not present in the sequences we examined.

**Coriell ID**	**Population**	**Nucleotide at Variant Positions in Coding Sequence**									
**for DNA**		**–135**	**47**	**190**	**552**	**629**	**754**	***7**	***44**	***313**	***315**
NA17347*3	AFRICANS SOUTH OF THE SAHARA	T T	C C	G G	A A	C C	A A	G G	G G	C C	T T
NA17344*A1	AFRICANS SOUTH OF THE SAHARA	T T	C C	G G	A A	T C	A A	G G	G G	C C	T T
NA10472*A2	BIAKA PYGMY POPULATION	T C	C C	G G	A A	T C	A A	A G	G G	C C	C C
NA07038*D2	CEPH/UTAH PEDIGREE 1333	T	C	G	A	C	A	G	G	C	C
NA11521*3	DRUZE POPULATION	T T	C A	G G	A A	T C	A A	A G	G G	C C	C C
NA17030*1	INDO PAKISTANI	T T	C C	T T	G G	C C	A A	G G	G G	C C	C C
NA10492*A2	MBUTI PYGMY POPULATION	T T	C C	G G	A A	C C	A A	G G	A G	C C	C T
NA10496*A3	MBUTI PYGMY POPULATION	T T	C C	G G	A A	C C	A A	G G	G G	C C	T T
NA17387*1	PACIFIC	C T	C C	G T	A G	C C	A A	G G	G G	C C	C C
NA13618*2	RUSSIAN - KRASNODAR	T T	C C	G G	A A	C C	A A	G G	G G	C C	T T
Frequency of Derived Nucleotide		0.11	0.05	0.16	0.16	0.16	0.00	0.11	0.05	0.00	0.47
