# Supplementary material for: NANOGP8: Evolution of a Human-Specific Retro-Oncogene
Source: G3 (Bethesda). 2012 Nov 1;2(11):1447–57. doi: 10.1534/g3.112.004366 (PMC3484675; doi:10.1534/g3.112.004366)
Supplement: Supporting Information [file supp_2.11.1447_FileS2.pdf]

File S2

Table S2 Genotypes at six variant positions in *NANOGP8* from single-pass sequences in exon 4 from 94 geographically diverse individuals

Coriell ID for DNA Sample	Population	Genotype at Variant Positions in Coding Sequence					
		552	629	754	916–917	*7	*44
NA17347*3	AFRICANS SOUTH OF THE SAHARA	hom A/A	hom C/C	hom A/A	hom TG/TG	hom G/G	hom G/G
NA17344*A1	AFRICANS SOUTH OF THE SAHARA	hom A/A	het C/T	hom A/A	hom TG/TG	hom A/A	hom G/G
NA10472*A2	BIAKA PYGMY POPULATION	hom A/A	het C/T	hom A/A	hom TG/TG	het G/A	hom G/G
NA07038*D2	CEPH/UTAH PEDIGREE 1333	hom A/A	hom C/C	het A/C	het TG/del	hom G/G	
NA10492*A2	MBUTI PYGMY POPULATION	hom A/A	hom C/C	hom A/A	hom TG/TG	hom G/G	het G/A
NA10496*A3	MBUTI PYGMY POPULATION	hom A/A	hom C/C	hom A/A	hom TG/TG	hom G/G	hom G/G
NA17387*1	PACIFIC	het A/G	hom C/C	hom A/A	hom TG/TG	hom G/G	hom G/G
NA13618*2	RUSSIAN - KRASNODAR	hom A/A	hom C/C	hom A/A	hom TG/TG	hom G/G	hom G/G
NA17348*1	AFRICANS SOUTH OF THE SAHARA	hom A/A	hom C/C	hom A/A	hom TG/TG	hom G/G	het G/A
NA17343*1	AFRICANS SOUTH OF THE SAHARA	hom A/A	hom C/C	hom A/A	hom TG/TG	hom G/G	het G/A
NA10473*A3	BIAKA PYGMY POPULATION	hom A/A	hom C/C	hom A/A	hom TG/TG	hom G/G	het G/A
NA17033*1	AFRICAN AMERICAN	hom A/A	hom C/C	hom A/A	hom TG/TG	hom G/G	hom A/A
NA17172*A1	AFRICAN AMERICAN	hom A/A	het C/T	het A/C	het TG/del	het G/A	
NA17161*A2	AFRICAN AMERICAN	hom A/A	het C/T	hom A/A	hom TG/TG	het G/A	hom G/G
NA17062*A1	MEXICAN	hom A/A	het C/T	hom A/A	hom TG/TG	het G/A	hom G/G
NA17036*A1	AFRICAN AMERICAN	hom A/A	het C/T	hom A/A	hom TG/TG	hom A/A	hom G/G
NA13617*1	RUSSIAN - KRASNODA	hom A/A	het C/T	hom A/A	hom TG/TG	hom A/A	hom G/G
NA17032*A1	AFRICAN AMERICAN	hom A/A	het C/T	hom A/A	hom TG/TG	hom A/A	hom G/G

NA10470*A3	BIAKA PYGMY POPULATION	hom A/A	het C/T	hom A/A	hom TG/TG	hom A/A	hom G/G
NA17342*2	AFRICANS SOUTH OF THE SAHARA	hom A/A	hom C/C	hom A/A	hom TG/TG	hom A/A	hom G/G
NA13609*1	AMI POPULATION	hom A/A	hom T/T	hom A/A	hom TG/TG	hom A/A	hom G/G
NA17636*1	MEXICAN-AMERICAN	hom A/A	hom T/T	hom A/A	hom TG/TG	hom A/A	hom G/G
NA17341*2	AFRICANS SOUTH OF THE SAHARA	hom A/A	hom T/T	hom A/A	hom TG/TG	hom A/A	hom G/G
NA17057*4	JAPANESE	hom A/A	hom T/T	hom A/A	hom TG/TG	hom A/A	hom G/G
NA17028*2	INDO PAKISTANI	het A/G	hom C/C	hom A/A	hom TG/TG	hom G/G	hom G/G
NA17317*A1	SOUTH AMERICA	het A/G	hom C/C	hom A/A	hom TG/TG	hom G/G	hom G/G
NA17076*2	PUERTO RICAN	het A/G	hom C/C	hom A/A	hom TG/TG	hom G/G	hom G/G
NA17065*A5	MEXICAN	het A/G	hom C/C	hom A/A	hom TG/TG	hom G/G	hom G/G
NA10849*5	CEPH/UTAH PEDIGREE 1332	het A/G	hom C/C	hom A/A	hom TG/TG	hom G/G	hom G/G
NA12911*2	CEPH/UTAH PEDIGREE 1582	het A/G	hom C/C	hom A/A	hom TG/TG	hom G/G	hom G/G
NA17313*A1	SOUTH AMERICA	het A/G	hom C/C	hom A/A	hom TG/TG	hom G/G	hom G/G
NA17040*A1	AFRICAN AMERICAN	hom A/A	hom C/C	hom A/A	hom TG/TG	hom G/G	hom G/G
NA17039*1	AFRICAN AMERICAN	hom A/A	hom C/C	hom A/A	hom TG/TG	hom G/G	hom G/G
NA17037*1	AFRICAN AMERICAN	hom A/A	hom C/C	hom A/A	hom TG/TG	hom G/G	hom G/G
NA17166*A1	AFRICAN AMERICAN	hom A/A	hom C/C	hom A/A	hom TG/TG	hom G/G	hom G/G
NA17345*2	AFRICANS SOUTH OF THE SAHARA	hom A/A	hom C/C	hom A/A	hom TG/TG	hom G/G	hom G/G
NA17346*2	AFRICANS SOUTH OF THE SAHARA	hom A/A	hom C/C	hom A/A	hom TG/TG	hom G/G	hom G/G
NA07057*D2	CEPH/UTAH PEDIGREE 1331	hom A/A	hom C/C	hom A/A	hom TG/TG	hom G/G	hom G/G
NA10858*B1	CEPH/UTAH PEDIGREE 1347	hom A/A	hom C/C	hom A/A	hom TG/TG	hom G/G	hom G/G
NA11993*C1	CEPH/UTAH PEDIGREE 1362	hom A/A	hom C/C	hom A/A	hom TG/TG	hom G/G	hom G/G
NA12909*2	CEPH/UTAH PEDIGREE 1477	hom A/A	hom C/C	hom A/A	hom TG/TG	hom G/G	hom G/G
NA17710*1	MEXICAN-AMERICAN	hom A/A	hom C/C	hom A/A	hom TG/TG	hom G/G	hom G/G
NA17443*2	MEXICAN-AMERICAN	hom A/A	hom C/C	hom A/A	hom TG/TG	hom G/G	hom G/G
NA18460*1	NOT IDENTIFIED	hom A/A	hom C/C	hom A/A	hom TG/TG	hom G/G	hom G/G

NA17072*A1	PUERTO RICAN	hom A/A	hom C/C	hom A/A	hom TG/TG	hom G/G	hom G/G
NA17071*A2	PUERTO RICAN	hom A/A	hom C/C	hom A/A	hom TG/TG	hom G/G	hom G/G
NA17314*2	SOUTH AMERICA	hom A/A	hom C/C	hom A/A	hom TG/TG	hom G/G	hom G/G
NA17088*2	SOUTHEAST ASIANS	hom A/A	hom C/C	hom A/A	hom TG/TG	hom G/G	hom G/G
NA12273*B2	CEPH/UTAH PEDIGREE 1418	hom A/A	hom C/C	hom A/A	hom TG/TG	hom G/G	hom G/G
NA11522*4	DRUZE POPULATION	hom A/A	hom C/C	hom A/A	hom TG/TG	hom G/G	hom G/G
NA11524*3	DRUZE POPULATION	hom A/A	hom C/C	hom A/A	hom TG/TG	hom G/G	hom G/G
NA17066*2	MEXICAN	hom A/A	hom C/C	hom A/A	hom TG/TG	hom G/G	hom G/G
NA17634*1	MEXICAN-AMERICAN	hom A/A	hom C/C	hom A/A	hom TG/TG	hom G/G	hom G/G
NA17311*A5	SOUTH AMERICA	hom A/A	hom C/C	hom A/A	hom TG/TG	hom G/G	hom G/G
NA06990*F1	CEPH/UTAH PEDIGREE 1331	hom A/A	hom C/C	hom A/A	hom TG/TG	hom G/G	hom G/G
NA17017*5	CHINESE (VERSION 1)	hom A/A	hom C/C	hom A/A	hom TG/TG	hom G/G	hom G/G
NA17016*3	CHINESE (VERSION 1)	hom A/A	hom C/C	hom A/A	hom TG/TG	hom G/G	hom G/G
NA17058*3	JAPANESE	hom A/A	hom C/C	hom A/A	hom TG/TG	hom G/G	hom G/G
NA17060*3	JAPANESE	hom A/A	hom C/C	hom A/A	hom TG/TG	hom G/G	hom G/G
NA17391*2	PACIFIC	hom A/A	hom C/C	hom A/A	hom TG/TG	hom G/G	hom G/G
NA10832*3	CEPH/UTAH PEDIGREE 1413	hom A/A	hom C/C	hom A/A	hom TG/TG	hom G/G	hom G/G
NA17389*2	PACIFIC	hom A/A	hom C/C	hom A/A	hom TG/TG	hom G/G	hom G/G
NA17388*1	PACIFIC	hom A/A	hom C/C	hom A/A	hom TG/TG	hom G/G	hom G/G
NA17073*A1	PUERTO RICAN	hom A/A	hom C/C	hom A/A	hom TG/TG	hom G/G	hom G/G
NA17056*B2	JAPANESE	hom A/A	hom C/C	hom A/A	hom TG/TG	hom G/G	hom G/G
NA10494*A3	MBUTI PYGMY POPULATION	hom A/A	hom C/C	hom A/A	hom TG/TG	hom G/G	hom G/G
NA17700*1	MEXICAN-AMERICAN	hom A/A	hom C/C	hom A/A	hom TG/TG	hom G/G	hom G/G
NA17075*2	PUERTO RICAN	hom A/A	hom C/C	hom A/A	hom TG/TG	hom G/G	hom G/G
NA17074*3	PUERTO RICAN	hom A/A	hom C/C	hom A/A	hom TG/TG	hom G/G	hom G/G
NA17315*1	SOUTH AMERICA	hom A/A	hom C/C	hom A/A	hom TG/TG	hom G/G	hom G/G

NA17087*2	SOUTHEAST ASIANS	hom A/A	hom C/C	hom A/A	hom TG/TG	hom G/G	hom G/G
NA17316*2	SOUTH AMERICA	hom A/A	hom C/C	hom A/A	hom TG/TG	hom G/G	hom G/G
NA17167*2	AFRICAN AMERICAN	hom A/A	hom C/C	hom A/A	hom TG/TG	hom G/G	hom G/G
NA07349*B1	CEPH/UTAH PEDIGREE 1345	hom A/A	hom C/C	hom A/A	hom TG/TG	hom G/G	hom G/G
NA10860*B1	CEPH/UTAH PEDIGREE 1362	hom A/A	hom C/C	hom A/A	hom TG/TG	hom G/G	hom G/G
NA10831*A7	CEPH/UTAH PEDIGREE 1408	hom A/A	hom C/C	hom A/A	hom TG/TG	hom G/G	hom G/G
NA10833*4	CEPH/UTAH PEDIGREE 1413	hom A/A	hom C/C	hom A/A	hom TG/TG	hom G/G	hom G/G
NA12813*5	CEPH/UTAH PEDIGREE 1454	hom A/A	hom C/C	hom A/A	hom TG/TG	hom G/G	hom G/G
NA12841*3	CEPH/UTAH PEDIGREE 1458	hom A/A	hom C/C	hom A/A	hom TG/TG	hom G/G	hom G/G
NA11523*3	DRUZE POPULATION	hom A/A	hom C/C	hom A/A	hom TG/TG	hom G/G	hom G/G
NA11525*3	DRUZE POPULATION	hom A/A	hom C/C	hom A/A	hom TG/TG	hom G/G	hom G/G
NA17067*A1	MEXICAN	hom A/A	hom C/C	hom A/A	hom TG/TG	hom G/G	hom G/G
NA17701*2	MEXICAN-AMERICAN	hom A/A	hom C/C	hom A/A	hom TG/TG	hom G/G	hom G/G
NA13611*2	AMI POPULATION	hom G/G	hom C/C	hom A/A	hom TG/TG	hom G/G	hom G/G
NA13607*5	AMI POPULATION	hom G/G	hom C/C	hom A/A	hom TG/TG	hom G/G	hom G/G
NA17349*3	AFRICANS SOUTH OF THE SAHARA	hom A/A	hom T/T	hom A/A	hom TG/TG	hom G/G	hom G/G
NA06987*D6	CEPH/UTAH PEDIGREE 1333	hom A/A	hom C/C	hom C/C	hom del	hom G/G	hom G/G
NA17158*3	AFRICAN AMERICAN	hom A/A	hom C/C	hom C/C	hom del	hom G/G	hom G/G
NA17035*A1	AFRICAN AMERICAN	hom A/A	het C/T	het A/C	het TG/del	het G/A	
NA17038*A1	AFRICAN AMERICAN	hom A/A	het C/T	het A/C	het TG/del	het G/A	
NA17061*A3	MEXICAN	hom A/A	hom C/C	het A/C	het TG/del	hom G/G	
NA17034*A2	AFRICAN AMERICAN	hom A/A	hom C/C	het A/C	het TG/del	hom G/G	
NA17078*1	PUERTO RICAN	hom A/A	hom C/C	het A/C	het TG/del	hom G/G	
NA10861*B3	CEPH/UTAH PEDIGREE 1362	het A/G	hom C/C	hom A/A	het TG/del	hom G/G	
Frequency of Derived Nucleotide		0.07	0.11	0.06	0.06	0.14	0.03
