# Supplementary material for: NANOGP8: Evolution of a Human-Specific Retro-Oncogene
Source: G3 (Bethesda). 2012 Nov 1;2(11):1447–57. doi: 10.1534/g3.112.004366 (PMC3484675; doi:10.1534/g3.112.004366)
Supplement: Supporting Information [file supp_2.11.1447_FileS3.pdf]

File S3

Table S3 Genotypes at two single-nucleotide substitution variant positions in *NANOG* from single-pass sequences in exon 4 from 94 geographically diverse individuals, and genotypes for the 22 bp deletion at position *552–*573, as determined by PCR analysis in 119 geographically diverse individuals.

**Coriell ID for DNA Sample**	**Population**	**Genotype at Variant Positions in Coding Sequence**		
		**531**	**798**	***552–*573**
NA17347*3	AFRICANS SOUTH OF THE SAHARA	het C/T	het C/T	het =/del
NA17344*A1	AFRICANS SOUTH OF THE SAHARA	hom C/C	het C/T	hom del/del
NA10472*A2	BIAKA PYGMY POPULATION	hom T/T	hom C/C	hom del/del
NA07038*D2	CEPH/UTAH PEDIGREE 1333	hom C/C	hom T/T	hom =/=
NA11521*3	DRUZE POPULATION			hom =/=
NA17030*1	INDO PAKISTANI			hom =/=
NA10492*A2	MBUTI PYGMY POPULATION	hom C/C	hom T/T	hom =/=
NA10496*A3	MBUTI PYGMY POPULATION	hom T/T	hom C/C	het =/del
NA17387*1	PACIFIC	hom T/T	hom C/C	hom =/=
NA13618*2	RUSSIAN - KRASNODAR	het C/T	het C/T	hom =/=
NA17348*1	AFRICANS SOUTH OF THE SAHARA	het C/T	het C/T	het =/del
NA17343*1	AFRICANS SOUTH OF THE SAHARA	het C/T	het C/T	het =/del
NA10473*A3	BIAKA PYGMY POPULATION	het C/T	het C/T	het =/del
NA17033*1	AFRICAN AMERICAN	het C/T	het C/T	het =/del
NA17172*A1	AFRICAN AMERICAN	het C/T	het C/T	het =/del
NA17161*A2	AFRICAN AMERICAN	hom T/T	hom C/C	hom del/del
NA17062*A1	MEXICAN	hom C/C	hom T/T	hom =/=

NA17036*A1	AFRICAN AMERICAN	het C/T	het C/T	het =/del
NA13617*1	RUSSIAN - KRASNODA	het C/T	het C/T	het =/del
NA17032*A1	AFRICAN AMERICAN	hom T/T	hom C/C	hom del/del
NA10470*A3	BIAKA PYGMY POPULATION	hom C/C	hom T/T	hom =/=
NA17342*2	AFRICANS SOUTH OF THE SAHARA	het C/T	het C/T	het =/del
NA13609*1	AMI POPULATION	het C/T	het C/T	het =/del
NA17636*1	MEXICAN-AMERICAN	het C/T	het C/T	het =/del
NA17341*2	AFRICANS SOUTH OF THE SAHARA	het C/T	het C/T	hom del/del
NA17057*4	JAPANESE	hom T/T	hom C/C	het =/del
NA17028*2	INDO PAKISTANI	het C/T	het C/T	hom =/=
NA17317*A1	SOUTH AMERICA	het C/T	het C/T	hom =/=
NA17076*2	PUERTO RICAN	het C/T	het C/T	hom del/del
NA17065*A5	MEXICAN	hom T/T	hom C/C	het =/del
NA10849*5	CEPH/UTAH PEDIGREE 1332	hom C/C	hom T/T	hom =/=
NA12911*2	CEPH/UTAH PEDIGREE 1582	hom C/C	hom T/T	hom =/=
NA17313*A1	SOUTH AMERICA	hom C/C	hom T/T	hom =/=
NA17040*A1	AFRICAN AMERICAN	het C/T	het C/T	het =/del
NA17039*1	AFRICAN AMERICAN	het C/T	het C/T	het =/del
NA17037*1	AFRICAN AMERICAN	het C/T	het C/T	het =/del
NA17166*A1	AFRICAN AMERICAN	het C/T	het C/T	het =/del
NA17345*2	AFRICANS SOUTH OF THE SAHARA	het C/T	het C/T	het =/del
NA17346*2	AFRICANS SOUTH OF THE SAHARA	het C/T	het C/T	het =/del
NA07057*D2	CEPH/UTAH PEDIGREE 1331	het C/T	het C/T	het =/del
NA10858*B1	CEPH/UTAH PEDIGREE 1347	het C/T	het C/T	het =/del
NA11993*C1	CEPH/UTAH PEDIGREE 1362	het C/T	het C/T	het =/del
NA12909*2	CEPH/UTAH PEDIGREE 1477	het C/T	het C/T	het =/del

NA17710*1	MEXICAN-AMERICAN	het C/T	het C/T	het =/del
NA17443*2	MEXICAN-AMERICAN	het C/T	het C/T	het =/del
NA18460*1	NOT IDENTIFIED	het C/T	het C/T	het =/del
NA17072*A1	PUERTO RICAN	het C/T	het C/T	het =/del
NA17071*A2	PUERTO RICAN	het C/T	het C/T	het =/del
NA17314*2	SOUTH AMERICA	het C/T	het C/T	het =/del
NA17088*2	SOUTHEAST ASIANS	het C/T	het C/T	het =/del
NA12273*B2	CEPH/UTAH PEDIGREE 1418	het C/T	het C/T	hom =/=
NA11522*4	DRUZE POPULATION	het C/T	het C/T	hom =/=
NA11524*3	DRUZE POPULATION	het C/T	het C/T	hom =/=
NA17066*2	MEXICAN	het C/T	het C/T	hom =/=
NA17634*1	MEXICAN-AMERICAN	het C/T	het C/T	hom =/=
NA17311*A5	SOUTH AMERICA	het C/T	het C/T	hom =/=
NA06990*F1	CEPH/UTAH PEDIGREE 1331	hom T/T	hom C/C	het =/del
NA17017*5	CHINESE (VERSION 1)	hom T/T	hom C/C	het =/del
NA17016*3	CHINESE (VERSION 1)	hom T/T	hom C/C	het =/del
NA17058*3	JAPANESE	hom T/T	hom C/C	het =/del
NA17060*3	JAPANESE	hom T/T	hom C/C	het =/del
NA17391*2	PACIFIC	hom T/T	hom C/C	het =/del
NA10832*3	CEPH/UTAH PEDIGREE 1413	hom T/T	hom C/C	hom =/=
NA17389*2	PACIFIC	hom T/T	hom C/C	hom =/=
NA17388*1	PACIFIC	hom T/T	hom C/C	hom =/=
NA17073*A1	PUERTO RICAN	hom T/T	hom C/C	hom =/=
NA17056*B2	JAPANESE	hom T/T	hom C/C	hom del/del
NA10494*A3	MBUTI PYGMY POPULATION	hom T/T	hom C/C	hom del/del
NA17700*1	MEXICAN-AMERICAN	hom T/T	hom C/C	hom del/del

NA17075*2	PUERTO RICAN	hom T/T	hom C/C	hom del/del
NA17074*3	PUERTO RICAN	hom T/T	hom C/C	hom del/del
NA17315*1	SOUTH AMERICA	hom T/T	hom C/C	hom del/del
NA17087*2	SOUTHEAST ASIANS	hom T/T	hom C/C	hom del/del
NA17316*2	SOUTH AMERICA	hom C/C	hom T/T	het =/del
NA17167*2	AFRICAN AMERICAN	hom C/C	hom T/T	hom =/=
NA07349*B1	CEPH/UTAH PEDIGREE 1345	hom C/C	hom T/T	hom =/=
NA10860*B1	CEPH/UTAH PEDIGREE 1362	hom C/C	hom T/T	hom =/=
NA10831*A7	CEPH/UTAH PEDIGREE 1408	hom C/C	hom T/T	hom =/=
NA10833*4	CEPH/UTAH PEDIGREE 1413	hom C/C	hom T/T	hom =/=
NA12813*5	CEPH/UTAH PEDIGREE 1454	hom C/C	hom T/T	hom =/=
NA12841*3	CEPH/UTAH PEDIGREE 1458	hom C/C	hom T/T	hom =/=
NA11523*3	DRUZE POPULATION	hom C/C	hom T/T	hom =/=
NA11525*3	DRUZE POPULATION	hom C/C	hom T/T	hom =/=
NA17067*A1	MEXICAN	hom C/C	hom T/T	hom =/=
NA17701*2	MEXICAN-AMERICAN	hom C/C	hom T/T	hom =/=
NA13611*2	AMI POPULATION	hom T/T	hom C/C	het =/del
NA13607*5	AMI POPULATION	hom T/T	hom C/C	hom del/del
NA17349*3	AFRICANS SOUTH OF THE SAHARA	hom C/C	hom T/T	hom =/=
NA06987*D6	CEPH/UTAH PEDIGREE 1333	het C/T	het C/T	het =/del
NA17158*3	AFRICAN AMERICAN	hom C/C	hom T/T	hom =/=
NA17035*A1	AFRICAN AMERICAN	het C/T	het C/T	hom del/del
NA17038*A1	AFRICAN AMERICAN	hom T/T	hom C/C	het =/del
NA17061*A3	MEXICAN	het C/T	het C/T	het =/del
NA17034*A2	AFRICAN AMERICAN	hom T/T	hom C/C	hom del/del
NA17078*1	PUERTO RICAN	hom T/T	hom C/C	hom del/del

NA10861*B3	CEPH/UTAH PEDIGREE 1362	het C/T	het C/T	het =/del
NA10469*B1	BIAKA PYGMY POPULATION			het =/del
NA10859*D3	CEPH/UTAH PEDIGREE 1347			het =/del
NA12749*3	CEPH/UTAH PEDIGREE 1444			het =/del
NA12912*3	CEPH/UTAH PEDIGREE 1582			het =/del
NA17018*A2	CHINESE			het =/del
NA17019*A2	CHINESE			het =/del
NA17063*2	MEXICAN			het =/del
NA17068*A2	MEXICAN			het =/del
NA17698*1	MEXICAN-AMERICAN			het =/del
NA17086*2	SOUTHEAST ASIANS			het =/del
NA07348*F1	CEPH/UTAH PEDIGREE 1345			hom =/=
NA17020*A1	CHINESE (VERSION 1)			hom =/=
NA17029*1	INDO PAKISTANI			hom =/=
NA10495*B1	MBUTI PYGMY POPULATION			hom =/=
NA17064*A2	MEXICAN			hom =/=
NA13619*2	RUSSIAN - KRASNODAR			hom =/=
NA17312*2	SOUTH AMERICA			hom =/=
NA13610*2	AMI POPULATION			hom del/del
NA13608*2	AMI POPULATION			hom del/del
NA10493*A1	MBUTI PYGMY POPULATION			hom del/del
NA17448*2	MEXICAN-AMERICAN			hom del/del
NA17684*1	MEXICAN-AMERICAN			hom del/del
NA17077*A1	PUERTO RICAN			hom del/del
