# Supplementary material for: NANOGP8: Evolution of a Human-Specific Retro-Oncogene
Source: G3 (Bethesda). 2012 Nov 1;2(11):1447–57. doi: 10.1534/g3.112.004366 (PMC3484675; doi:10.1534/g3.112.004366)
Supplement: Supporting Information [file supp_2.11.1447_FileS4.pdf]

File S4

Evidence of Two Major Haplotypes of *NANOG* Throughout Human Populations Worldwide, and Intragenic Recombination Between Haplotypes

Sequences we obtained of *NANOG* exon 4 from 94 geographically diverse individuals revealed two highly polymorphic synonymous substitution variants, *c.531C>T* and *c.798C>T*, in the reading frame. In both cases, C is ancestral and T derived. In *NANOGP8*, the ancestral nucleotides at both positions were homozygous in all 94 individuals, evidence that the parent allele of *NANOG* at the time of *NANOGP8*'s origin carried the ancestral nucleotides at both sites. We were able to distinguish homozygotes and heterozygotes for these two substitution polymorphisms in all 94 individuals tested, and compared them to the genotypes previously determined for the **552–*573del* deletion. As shown in Table S4, of the 27 possible genotypes for these three polymorphisms, only nine were observed.

Hereafter, and in Table S4, we denote haplotypes for these variants as abbreviations in accordance with current human haplotype nomenclature (http://www.hgvs.org/mutnomen) as follows: For positions c.531 and c.798, the nucleotide is listed (C or T). For position c.*552–*573, = denotes the ancestral non-deleted sequence, and *del* the derived deleted sequence. Semicolons separate variants at these three sites in their respective order.

Haplotypes could be conclusively identified only in triple homozygotes and individuals heterozygous for one of the three variants, a total of 51 individuals. The two most prevalent haplotypes are the reciprocal haplotypes *T*; *C*; *del* and *C*; *T*; =, which, respectively, are the two haplotypes in the current primary and alternate genomic assemblies of *NANOG*. The third most frequent haplotype is *T*; *C*; =, which probably arose through intragenic recombination between the two most prevalent haplotypes. Its reciprocal haplotype, *C*; *T*; *del*, also an apparent intragenic recombinant, is rare in the samples we examined, confirmed to be present in the heterozygous condition in five individuals. Only one individual (from the Africans South of the Sahara panel) carries as a heterozygote the fifth haplotype, *C*; *C*; *del*, which is the ancestral parent haplotype of *NANOGP8*. As shown in Table S4, double and triple heterozygotes could best be explained as genotypes heterozygous for the four most prevalent haplotypes; alternative genotypes for double and triple heterozygotes require the *T*; *T*; = or *T*; *T*; *del* haplotypes, which were not observed in any triple homozygotes or single heterozygotes.

Minor allele frequencies (MAFs) in the *NANOG* NCBI dbSNP report for five variants in the reading frame (*c.165C>T*, *c.246T>G*, *c.276G>A*, *c.531C>T*, and *c.798C>T*) are essentially equal, ranging from 0.3768 to 0.3863. In all five cases, the minor allele (two of which are ancestral and three derived) is the nucleotide present in the alternate

reference assembly, which corresponds to haplotype *C; T; =* in our sequences. Also, for each of these five variants, the major allele is the nucleotide present in the primary reference assembly (three ancestral and two derived), which corresponds to haplotype *T; C; del* in our sequences. These observations suggest that the *NANOG* sequences in the primary and alternate assemblies represent two major haplotypes that differ by five substitution variants within the reading frame. They correspond, respectively, to alleles *a* and *b* of *NANOG* identified by Zbiden *et al.* (2010). We used the reading-frame sequence of the primary and alternate assemblies of *NANOG* as a query for MEGABLAST searches (using default parameters) against the human EST database and identified 21 ESTs with 98% or greater similarity spanning at least two of these variant sites. Fifteen of these ESTs had a sequence consistent with the primary-assembly haplotype and six with the alternate-assembly haplotype, with no identifiable intragenic recombinants within the reading frame, further evidence of two prevalent haplotypes for *NANOG*.

Table S4 A. Observed genotypes and inferred haplotypes in *NANOG* for the *c.531C>T*, *c.798C>T*, and **552–*573del* derived variants and their ancestral counterparts (=).

**Observed Genotype**	**Number of Individuals**	**Inferred Haplotypes**
Triple homozygote *C/C; T/T; =/=*	20	*C; T; =*
Triple homozygote *T/T; C/C; del/del*	13	*T; C; del*
Triple homozygote *T/T; C/C; =/=*	5	*T; C; =*
Single heterozygote *T/T; C/C; =/del*	11	*T; C; =/T; C; del*
Single heterozygote *C/C; T/T; =/del*	1	*C; T; =/C; T; del*
Single heterozygote *C/C; C/T; del/del*	1	*C; T; del/C; C; del*
Double heterozygote *C/T; C/T; =/=*	9	*C; T; =/T; C; =* (most probable)
Double heterozygote *C/T; C/T; del/del*	3	*T; C; del/C; T; del* (most probable)
Triple heterozygote *C/T; C/T; =/del*	31	*C; T; =/ T; C; del* (most probable)
**Total**	**94**	

B. Number and frequency of chromosomes with inferred haplotypes

Haplotype	Number of Chromosomes	Frequency
C; T; = (major haplotype, alternate assembly)	81	0.4309
T; C; del (major haplotype, primary assembly)	71	0.3777
T; C; = (recombinant haplotype)	30	0.1596
C; T; del (recombinant haplotype)	5	0.0266
C; C; del (ancestral haplotype)	1	0.0053
