# Supplementary material for: NANOGP8: Evolution of a Human-Specific Retro-Oncogene
Source: G3 (Bethesda). 2012 Nov 1;2(11):1447–57. doi: 10.1534/g3.112.004366 (PMC3484675; doi:10.1534/g3.112.004366)
Supplement: Supporting Information [file supp_2.11.1447_FileS5.pdf]

File S5

An Analysis of Previously Published Efforts to Distinguish RT-PCR Products Derived from *NANOG* and *NANOGP8* in Cancer Cells

Because both *NANOG* and *NANOGP8* may be transcriptionally active in cancer cells, accurate distinction of their RT-PCR products is essential for gene-expression research. The genomic differences between *NANOG* and *NANOGP8* are considerably greater than those in RT-PCR products, due to the presence of introns in *NANOG* and their absence in *NANOGP8*, and different flanking sequences at the genomic borders of *NANOG* and *NANOGP8* the insertion boundaries of *NANOGP8*. Ultimately, RT-PCR products must be distinguished based on variants within the mRNA that differ between the two in the cell cultures under study. Researchers who have studied differential expression of *NANOG* and *NANOGP8* in cancer cells have relied on a variety of differences between *NANOG* and *NANOGP8* sequences in genomic reference assemblies for experimental distinction of RT-PCR products. According to our experimental results, however, differences in reference assemblies may be unreliable because they may represent modern polymorphisms present in a subset of individuals.

Zhang et al. (2006) published the first evidence that *NANOGP8* is a retrogene expressed in cancer cells. They utilized primers capable of amplifying RT-PCR products from both *NANOG* and *NANOGP8*, but distinguished the two through sequencing the reading frame of their RT-PCR products. The sequences they identified as belonging to *NANOGP8* contained variants we identified as evidently fixed in *NANOGP8* (*c.144G>A* and *c.759G>C*), confirming the accuracy of their identifications. Likewise, Zhang et al. (2010) and Uchino et al. (2012) utilized primers capable of amplifying RT-PCR products from both *NANOG* and *NANOGP8* and sequenced their RT-PCR products confirming their correct identities.

Jeter et al. (2009, 2011), Ma et al. (2010, 2012), and Ibrahim et al. (2012) relied on the 22-nucleotide pair deletion in the 3´ UTR of *NANOGP8* (c.*552_*573del) as a site for primers and probes to distinguish *NANOG* from *NANOGP8* qRT-PCR products, presuming this deletion to be unique to *NANOGP8* based on an earlier human genome reference assembly. Our results demonstrate that this deletion is uniformly present in *NANOGP8* but highly polymorphic in *NANOG*. Its use as a primer-binding site for RT-PCR should result in reliable amplification of *NANOGP8* fragments but may also result in amplification of fragments of identical size from *NANOG*, if *NANOG* is transcribed in those

cells and if the cells are from individuals who carry the *c.*552_*573del* allele of *NANOG* (a majority of individuals according to our results). Jeter et al. (2009) and Ibrahim *et al.* (2012) sequenced RT-PCR products they obtained, confirming the correct identifications of these products as belonging to *NANOGP8* or *NANOG* on the basis of reading-frame variants that, according to our research, are evidently fixed. There is no indication of sequencing to confirm correct identification of RT-PCR products in other studies utilizing *c.*552_*573del* as primer-binding site (Jeter et al. 2011, Ma et al. 2010, 2012).

Ambady et al. (2010) relied on a *Sma*I RFLP generated by a derived substitution variant in the 3´ UTR (*c*313C>G*) to distinguish *NANOG* and *NANOGP8* RT-PCR products, presuming the *Sma*I site to be unique to *NANOG* based on reference-sequence comparison. Our results, however, demonstrate that the ancestral *Sma*I site and the derived variant in *NANOGP8* that alters the site to create the RFLP are highly polymorphic in *NANOGP8*, rendering this RFLP unreliable for distinguishing *NANOG* and *NANOGP8* RT-PCR products. However, Ambady et al. (2010) sequenced the RT-PCR products they obtained, confirming their correct identity as *NANOGP8*.

Zbinden et al. (2010) sequenced a region they referred to as "a diagnostic 3´ UTR region, which varies among the *NANOG* alleles and *NANOGP8*" (p. 2660), based on their comparison of reference sequences. However, they did not specify which variants they considered as diagnostic. According to our results, all variants in the 3´ UTR are modern polymorphisms in either *NANOG* or *NANOGP8*, except **606T>G* in *NANOGP8*, which we could not confirm as fixed or polymorphic.

Eberle et al. (2010) utilized RT-PCR to detect *NANOG* transcripts in acute leukemic human cell lines, and concluded that *NANOGP8* was not expressed in these cells. Their conclusion was based on two primer pairs (which they named set a and set b) presumed to amplify fragments from transcripts of both *NANOG* and *NANOGP8*, as well as other *NANOG* pseudogenes. They sequenced the RT-PCR products from these primer pairs but did not state which variants they considered to be reliable identifiers of *NANOG* and *NANOGP8*. One of their primer pairs (set a) should have amplified fragments of identical size from both *NANOG* and *NANOGP8* transcripts, consisting of most of the reading frame. The reverse primer of the other pair (set b), however, had on its 3´ end the ancestral G at site c.759, which is present in *NANOG*, but altered in *NANOGP8* by the *c.759G>C* fixed variant. Therefore, this primer pair should have successfully amplified fragments from *NANOG* but not *NANOGP8*. To confirm their conclusion that *NANOGP8* was not expressed in these cells, they utilized a third primer pair they considered to be exclusive to *NANOGP8*, and detected no amplification in any of 60 clones from a single cell line. This primer pair, however, relied on the *c.47C>A* variant on the 3´ end of the forward primer for exclusive amplification of *NANOGP8*. Although this derived variant is present in both the current primary and alternate reference assemblies of *NANOGP8*, our results, as well as those of Jeter et al. (2009) and Uchino et al. (2012), show it to be polymorphic

and rare in *NANOGP8*. Moreover, the reverse primer in this pair had the derived T in the *c.531C>T* variant as the third nucleotide from the 3′ end of the primer, which is absent in *NANOGP8* and polymorphic in *NANOG*, according to our sequences. Therefore, this primer pair, considered to be *NANOGP8*-specific, is not likely to amplify fragments from either *NANOGP8* or *NANOG* in most individuals, and may explain the lack of amplification observed. Beyond these issues for *NANOGP8* identification, the results of Eberle et al. (2010) are highly pertinent in that alternative splicing of transcripts from *NANOG* and *NANOGP1* was well documented in these cell lines.

Ishiguro et al. (2012) relied on an RFLP resulting from the *c.144G>A* variant in *NANOGP8* to distinguish *NANOG* and *NANOGP8* RT-PCR products. Our data suggest that this is a reliable variant due to evident fixation of the ancestral allele in *NANOG* and the derived allele in *NANOGP8*.

Our observations of widespread modern polymorphisms in *NANOG* and *NANOGP8* underscore the unreliability of variants between reference sequences for accurate experimental identification of RT-PCR products. Instead, the most reliable method to distinguish *NANOG* from *NANOGP8* RT-PCR products is to sequence genomic-DNA specific to *NANOG* and *NANOGP8* from the cell lines being researched to identify which variants distinguish the two in any particular cell line, then use those variants to accurately identify sequenced RT-PCR products for each line. Several of the primer pairs we used (see Table 1 in the main text of the article) are capable of generating PCR fragments specific to *NANOG* or *NANOGP8* from genomic DNA (albeit not from mRNA for RT-PCR), and may be useful for such genomic-DNA sequencing.
